# Controlling Cation Distribution and Morphology in Colloidal Zinc Ferrite Nanocrystals

**DOI:** 10.1021/acs.chemmater.2c01568

**Published:** 2022-08-01

**Authors:** Karla R. Sanchez-Lievanos, Kathryn E. Knowles

**Affiliations:** Department of Chemistry, University of Rochester, Rochester, New York 14627, United States

## Abstract

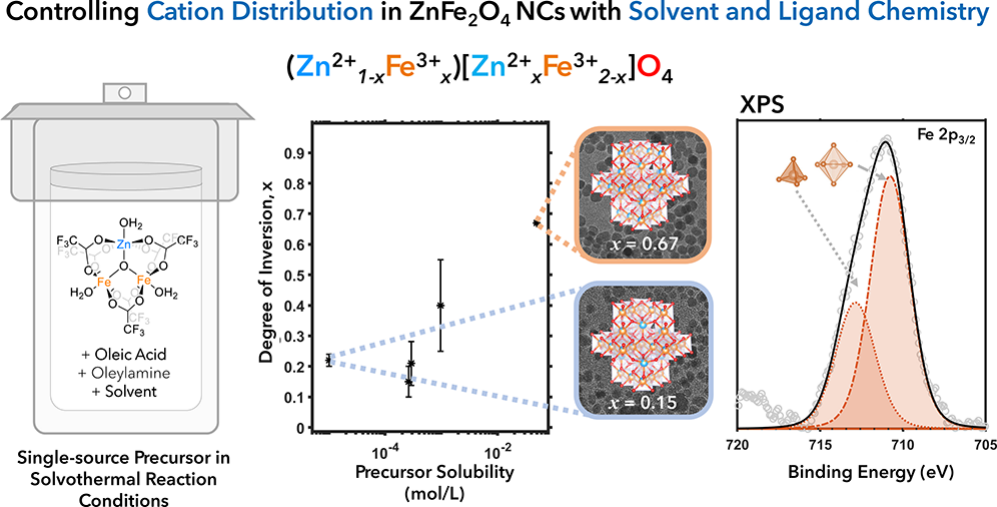

This paper describes the first synthetic method to achieve independent control over both the cation distribution (quantified by the inversion parameter *x*) and size of colloidal ZnFe_2_O_4_ nanocrystals. Use of a heterobimetallic triangular complex of formula ZnFe_2_(μ_3_-O)(μ_2_-O_2_CCF_3_)_6_(H_2_O)_3_ as a single-source precursor, solvothermal reaction conditions, absence of hydroxyl groups from the reaction solvent, and the presence of oleylamine are required to achieve well-defined, crystalline, and monodisperse ZnFe_2_O_4_ nanoparticles. The size of the ZnFe_2_O_4_ nanocrystals increases as the ratio of oleic acid and oleylamine ligands to precursor increases. The inversion parameter increases with increasing solubility of the precursor in the reaction solvent, with the presence of oleic acid in the reaction mixture, and with decreasing reaction temperature. These results are consistent with a mechanism in which ligand exchange between oleic acid and carboxylate ligands bound to the precursor complex influences the degree to which the reaction produces a kinetically trapped or thermodynamically stable cation distribution. Importantly, these results indicate that preservation of the triangular Zn–O–Fe_2_ core structure of the precursor in the reactive monomer species is crucial to the production of a phase-pure ZnFe_2_O_4_ product and to the ability to tune the cation distribution. Overall, these results demonstrate the advantages of using a single-source precursor and solvothermal reaction conditions to achieve synthetic control over the structure of ternary spinel ferrite nanocrystals.

## Introduction

Transition-metal oxide nanocrystals are attractive for many different applications in energy conversion, storage, and photocatalysis due to their generally excellent chemical and thermal stability and their high surface area-to-volume ratio.^1−4^ To advance the fundamental understanding and ultimate application of these nanomaterials, it is essential to develop synthetic methods that provide access to high-quality samples with controlled size, crystal structure, composition, and morphology.^5^ Over the last two decades, heat-up and hot-injection methods involving the reaction of precursors at elevated temperature and ambient pressure in high-boiling organic solvents have evolved to provide exquisite control over noble metal and metal chalcogenide semiconductor nanocrystals.^6−8^ Ternary and alloyed materials with controlled compositions,^9^ core–shell structures,^10,11^ and exceptionally high monodispersity in both isotropic and anisotropic morphologies^12−14^ are all accessible for these materials. In contrast, transition-metal oxide nanomaterials, particularly ternary metal oxide nanocrystals, often cannot be made using these well-developed colloidal techniques. Instead, these nanocrystals are generally made using sol–gel,^15^ electrochemical,^16^ microwave,^17^ or solvothermal methods.^18,19^ Such methods often provide only crude means of morphology control and usually produce nanocrystals that are not stable as colloidal dispersions.^20,21^ Developing solvothermal methods that provide the same level of synthetic control over ternary oxide nanocrystals that heat-up or hot-injection approaches have provided to metal and metal chalcogenide nanocrystals is crucial for the development of next-generation metal oxide nanomaterials.

Spinel ferrite nanocrystals are a class of ternary oxide materials that have recently shown promising photocatalytic activity under visible light for hydrogen production,^22^ water oxidation,^23^ and advanced oxidative degradation of organic pollutants for water remediation.^24−26^ These materials have the general formula (M_1–*x*_Fe_*x*_)[M_*x*_Fe_2–*x*_]O_4_, where M is a divalent metal cation, and the parentheses and square brackets represent tetrahedral and octahedral cation sites within the spinel crystal structure, respectively. The inversion parameter, *x*, quantifies the distribution of M^2+^ and Fe^3+^ cations among these two types of crystallographic sites. This structural parameter significantly impacts not only the electronic, optical, and magnetic properties of a spinel ferrite material^27^ but also has been both proposed^28^ and observed^17^ to impact its photocatalytic activity. In bulk spinel ferrites, the thermodynamically favored magnitude of *x* depends on several factors such as the relative size of the two cations and the relative crystal field stabilization energies.^27,29,30^ Increased structural degrees of freedom provided by large surface area-to-volume ratios in nanoscale spinel ferrites broaden the range of accessible cation distributions in these materials.^27^ Developing synthetic methods for tuning the inversion parameter along with the size, shape, and composition of spinel ferrite nanocrystals would provide another dimension of control over the properties and performance of these materials.

Recently, our group reported the use of heterobimetallic trinuclear molecular complexes with the formula MFe_2_(μ_3_-O)(μ_2_-O_2_CR)_6_(H_2_O)_3_ as single-source precursors for the solvothermal synthesis of a series of spinel ferrite nanocrystals of formula MFe_2_O_4_, where M = Fe, Co, Ni, Cu, and Zn.^18^ This work demonstrates that the use of a single-source precursor produces more monodisperse nanocrystals with improved phase purity compared to nanocrystals synthesized from a mixture of multisource precursors. Here, we build on this initial report by identifying reaction conditions that influence the size, shape, and cation distribution of spinel ferrite nanocrystals synthesized from these single-source precursors. We use ZnFe_2_O_4_ as a model material because it combines a diamagnetic ion (Zn^2+^) with a paramagnetic ion (Fe^3+^) and its magnetic properties are therefore particularly sensitive to the cation distribution.^31−34^ Additionally, ZnFe_2_O_4_ efficiently absorbs visible light and has band-edge positions that are capable of driving advanced oxidation processes, such as organic pollutant degradation in water.^35−37^ It is thus an outstanding candidate for applications in energy conversion, targeted drug delivery, and photocatalysis.^38−41^

This paper describes a comprehensive set of systematic studies that provide important insights into the synthesis of colloidal spinel ZnFe_2_O_4_ nanocrystals using the single-source precursor ZnFe_2_(μ_3_-O)(μ_2_-O_2_CCF_3_)_6_(H_2_O)_3_ under solvothermal reaction conditions. We demonstrate that subjecting the same reaction mixtures to colloidal heat-up or hot-injection procedures, conducted at ambient pressure on a Schlenk line, produces largely amorphous and polydisperse nanoparticles. We find that varying the concentration and chemical structure of free ligands added to the solvothermal reaction impacts the size and monodispersity of the resulting ZnFe_2_O_4_ nanocrystals. These studies provide empirical guidelines for the synthesis of monodisperse ZnFe_2_O_4_ nanocrystals for which the average nanoparticle size can be tuned by changing the precursor-to-ligand ratio. We also show that changing the solvent used for the reaction impacts both the crystal phase and the cation distribution. Solvents that contain hydroxyl groups, such as alcohols, polyols, and water, produce mixtures of nanocrystals with a variety of compositions including the desired spinel ZnFe_2_O_4_ phase, as well as binary oxide phases such as α-Fe_2_O_3_ and ZnO. In contrast, aromatic solvents, without hydroxyl groups, produce phase-pure ZnFe_2_O_4_ nanocrystals with inversion parameters that vary from *x* = 0–0.67 depending on the solubility of the precursor in the reaction solvent and the presence of oleic acid in the reaction. Based on these results, we propose a mechanism in which the oxo-bridged Zn–O–Fe_2_ core of the precursor molecule remains intact in the reactive monomer species that participates in nanocrystal nucleation and growth. The exchange of bridging trifluoroacetate ligands with oleic acid modulates the kinetics of precursor conversion and nucleation steps and thereby mediates the ability of the reaction system to access a thermodynamically stable or kinetically trapped cation distribution.

## Experimental Section

### Materials

Iron(III) nitrate nonahydrate (Fe(NO_3_)_3_·9H_2_O, >98%), zinc(II) nitrate hexahydrate (Zn(NO_3_)_2_·6H_2_O, >98%), trifluoroacetic acid (99%), oleic acid (90%), hexanoic acid (99%), lauric acid (≥98%), oleylamine (≥98%), hexadecylamine (98%), dodecylamine (≥99%), benzene (99%), toluene (99.5%), dibenzyl ether (99%), phenyl ether (98%), catechol (≥99%), glycerol (≥99%), ethylene glycol (≥99%), and tetrachloroethylene (≥99.5%) were purchased from Sigma-Aldrich. Phenol (≥99%) and sodium hydroxide (98%) were purchased from Fisher Scientific. The above-mentioned chemicals were used as received without further purification. *Caution*! Trifluoroacetic acid is both volatile and corrosive and should therefore be handled exclusively in a fume hood.

### Synthesis of ZnFe_2_(μ_3_-O)(μ_2_-O_2_CCF_3_)_6_(H_2_O)_3_·4C(O)Me_2_·H_2_O (**1**)

The oxo-centered triangular cluster was prepared according to our previously reported synthesis procedures.^18^ Briefly, Fe(NO_3_)_3_·9H_2_O (1.387 g, 3.4 mmol) and Zn(NO_3_)_2_·6H_2_O (0.516 g, 1.7 mmol) were dissolved separately in two vials each containing 5 mL of Nanopure water. NaOH (0.489 g, 12 mmol) was dissolved in 10 mL of Nanopure water and mixed with trifluoroacetic acid (4.188 g, 36 mmol in 10 mL of nanopure water) in a 250 mL round-bottom flask. The metal nitrate salt solutions were subsequently added to the resulting solution of sodium trifluoroacetate, and the reaction mixture was heated to 85 °C under an ambient atmosphere and left stirring for 20 h until a homogeneous solution formed. After cooling, excess water and trifluoroacetic acid were removed under rotary evaporation. The remaining solid product was dissolved in cold acetone or acetonitrile and separated from sodium nitrate via vacuum-assisted filtration. This process was repeated three times. The resulting filtrate was dried at 45 °C under vacuum for at least 1 h, yielding a fine powder. This powder was again dissolved in HPLC-grade acetone or acetonitrile and crystallized over a period of 48 h inside a refrigerator at 4 °C. The crystals were filtered, dried at 45 °C under vacuum, and used in the nanocrystal synthesis without further workup. The cluster was synthesized on average in a good yield (83%). UV–vis (λ_max_[nm] (ε[M^–1^ cm^–1^])): 234 (5973), 315 (2684), 340 (2166), 465 (21). FTIR (cm^–1^): 640, 696.3, 725.2, 794.7, 854.5, 1150, 1196, 1346, 1474, 1649, 1682. ^19^F NMR (δ ppm): −35 and −53. EA (%): C 17.55; H 1.32; N 0.33 consistent with ZnFe_2_C_12_F_18_H_6_O_16_·C_3_H_6_O and trace amounts of sodium nitrate salts.

### Synthesis of ZnFe_2_O_4_ Nanocrystals under Solvothermal Conditions

In general, **1** (0.0231 g, 0.025 mmol), oleylamine (OAm, 0.737 g, 2.7 mmol based on 98% purity), oleic acid (OA, 0.848 g, 2.7 mmol based on 90% purity), and solvent (10 mL) were added to a 25 mL Teflon insert. The mixture was stirred for 15 min under ambient conditions to form a clear dark red suspension. Subsequently, the Teflon insert was loaded into a stainless-steel autoclave, sealed, and heated at 230 °C for 24 h. The autoclave was allowed to cool down over a period of 8–12 h under a well-ventilated fume hood. The suspension was then purified with three cycles of precipitation with ethanol followed by centrifugation. These conditions correspond to a **1**:OA:OAm ratio of 1:108:108 and were used for all of the reactions that varied the reaction solvent. Additional details for reactions in which the precursor-to-ligand ratio varied from 1:108:108 are provided in Table S4.

### Nanocrystal Characterization

#### Powder X-ray Diffraction (XRD)

We performed powder XRD measurements on dried nanocrystal powders using a Rigaku XtaLAB Dualflex Synergy-S diffraction system with Mo Kα radiation (λ = 0.71073 Å). We converted the 2θ values obtained using the Mo source to 2θ values corresponding to the wavelength of a Cu Kα source (λ = 1.54148 Å) to compare our measured spectra to standard data deposited in the JCPDS database that was collected with Cu Kα radiation.

#### Transmission Electron Microscopy (TEM)

TEM micrographs and selected area electron diffraction (SAED) patterns were obtained using an FEI Tecnai F20 TEM with a beam energy of 200 kV. The nanocrystal samples were drop-casted onto lacey carbon copper grids from hexane dispersions. The diameter of the particles was measured using ImageJ software.^42^

#### X-ray Photoelectron Spectroscopy (XPS)

XPS measurements were performed on three separate samples of each nanocrystal batch to ensure data reproducibility. Sample preparation was performed under an ambient atmosphere. The nanocrystal powders were dissolved in hexane to obtain a concentrated solution. The solution was drop-casted onto cleaned Si wafers, which were electrically grounded to the sample bar by carbon tape. The XPS measurements were recorded with a Kratos Axis Ultra DLD system equipped with a monochromatic Al Kα (*h*ν = 1486.6 eV) X-ray source. During the measurements, pressure in the main chamber was kept below 5 × 10^–7^ mbar. Charge compensation was carried out via a neutralizer running at a current of 7 × 10^–6^ A, a charge balance of 5 eV, and a filament bias of 1.3 V. The X-ray gun was set to 10 mA emission. Binding energies were referenced to the C 1s peak arising from adventitious carbon with a binding energy of 284.8 eV. The C 1s and Fe 2p core levels were recorded with a pass energy of 20 eV. We collected five scans for iron and two scans for carbon. XPS analysis was performed with CasaXPS (Version 2.3.22PR1.0.)^43^ The U Touggard function was used for background subtraction. The Fe 2p_3/2_ XPS signals were fitted with the CasaXPS Component Fitting tool.

#### Energy-Dispersive X-ray Spectroscopy (EDS)

Elemental compositions of nanocrystal samples were analyzed using a Zeiss Auriga Scanning Electron Microscope coupled to an energy-dispersive X-ray spectroscopy (EDS) analyzer. Measurements were carried out using 25 kV electron beam energy. Semiquantitative data analyses were performed using the energy-dispersive X-ray analysis (EDAX) Apex software.

## Results

### Solvothermal Reaction Conditions are Required to Achieve Monodisperse Colloidal ZnFe_2_O_4_ Nanocrystals

**Figure 1:**
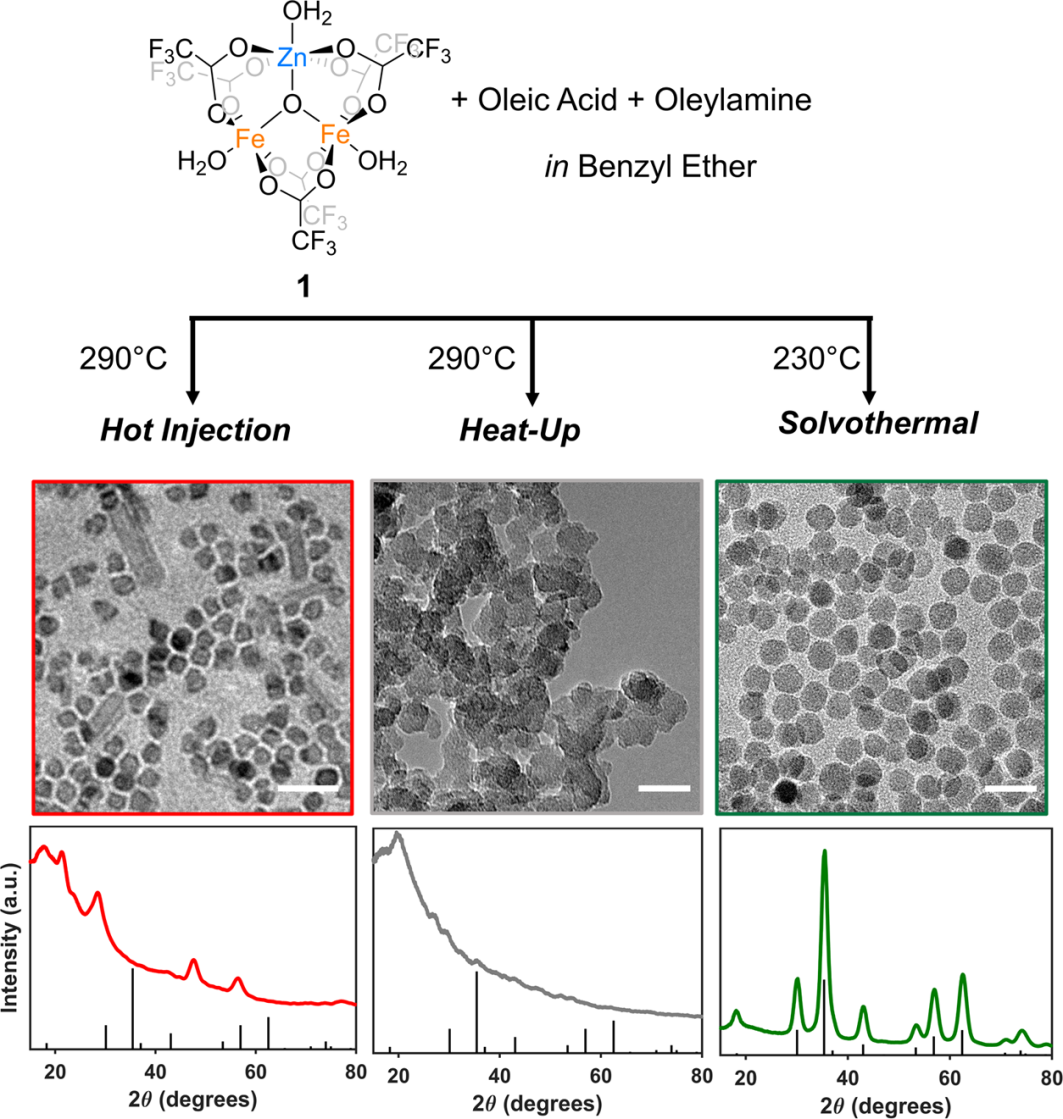
Top: Reaction scheme for the syntheses of ZnFe_2_O_4_ NCs via hot-injection, heat-up, and solvothermal reactions of cluster **1** in the presence of oleic acid and oleylamine as surfactants and benzyl ether as the solvent. Middle and Bottom: Representative bright-field TEM micrographs and powder X-ray diffractograms of products obtained from these reactions. The scale bars represent 20 nm. The black stems on the diffractograms are the standard reference peaks for the spinel zinc ferrite crystal phase (JCPDS Card No. 01-089-1009).

Our previous work demonstrated that trinuclear heterobimetallic single-source precursors of formula MFe_2_(μ_3_-O)(μ_2_-O_2_CR)_6_(H_2_O)_3_ (M = Fe, Co, Ni, Cu, and Zn, R = CF_3_) produce more monodisperse and phase-pure spinel ferrite nanocrystals than mixtures of multisource metal salt precursors; however, this work only explored solvothermal reaction conditions, namely, benzyl ether as a reaction solvent at 230 °C in the presence of oleic acid and oleylamine.^18^ To develop methods to control the size, shape, and cation distribution of these nanocrystals, we must first understand the mechanisms by which the cluster precursors nucleate and grow spinel ferrite nanocrystals. Hot-injection and heat-up reactions conducted at ambient pressure are much more conducive to mechanistic studies than solvothermal reactions due to the ability to extract aliquots during the reaction. Although hot-injection and heat-up methods have been used to synthesize ternary spinel ferrite nanocrystals from single-source precursors with structures similar to **1**,^44−47^ to date, these methods have not yielded a detailed mechanistic understanding of the nanocrystal formation process. Inspired to improve on these reports, we attempted to synthesize ZnFe_2_O_4_ nanocrystals by subjecting mixtures of **1**, oleic acid, and oleylamine to hot-injection and heat-up reaction conditions. We used the same reaction mixture across all methods. For the hot-injection reactions, **1** was dissolved in benzyl ether and injected into a solution containing benzyl ether, oleic acid, and oleylamine at the reaction temperature (230 °C). For the heat-up reaction, we added **1** dissolved in benzyl ether, oleic acid, and oleylamine to a three-neck flask equipped with a reflux condenser and heated the solution to 230 °C. After heating for 60 min at 230 °C, no color changes were observed, and no nanoparticles were recovered from either the heat-up or hot-injection reactions (see the Supporting Information). Performing hot-injection and heat-up reactions at 290 °C, closer to the boiling point of benzyl ether (298 °C), produced nanoparticles with poor or negligible crystallinity and polydisperse morphologies (Figure 1). In contrast, subjecting the same mixtures to solvothermal reaction conditions, involving the use of an autoclave reactor at 230 °C, produced high-quality, crystalline, and phase-pure ZnFe_2_O_4_ nanocrystals (Figure 1). These results indicate that the elevated pressure achieved in an autoclave reactor under solvothermal conditions is necessary for the formation of high-quality spinel ZnFe_2_O_4_ nanocrystals from the single-source precursor, **1**, at the concentrations used here. We note that crystalline MFe_2_O_4_ nanoparticles have been obtained from heat-up reactions using similar single-source precursors to **1**, but at precursor concentrations that range from 3 to 30 times larger than that used here.^45−47^ Analogously, crystalline ternary spinel oxide minerals are naturally formed under geological conditions that access elevated pressure.^48^ We also suspect that the elevated pressure of the solvothermal reactions enables higher concentrations of water to remain in the reaction mixture throughout the reaction duration. As described in the discussion section and demonstrated by our previous work,^49,50^ we hypothesize that water is necessary for the hydrolysis reactions that lead to metal oxide formation. The heat-up and hot-injection reactions run at 290 °C under ambient pressure are more likely to drive water into the vapor phase where it is less effective at promoting metal oxide formation.

### Surfactant Ligands Influence the Size and Polydispersity of ZnFe_2_O_4_ Nanocrystals

**Figure 2:**
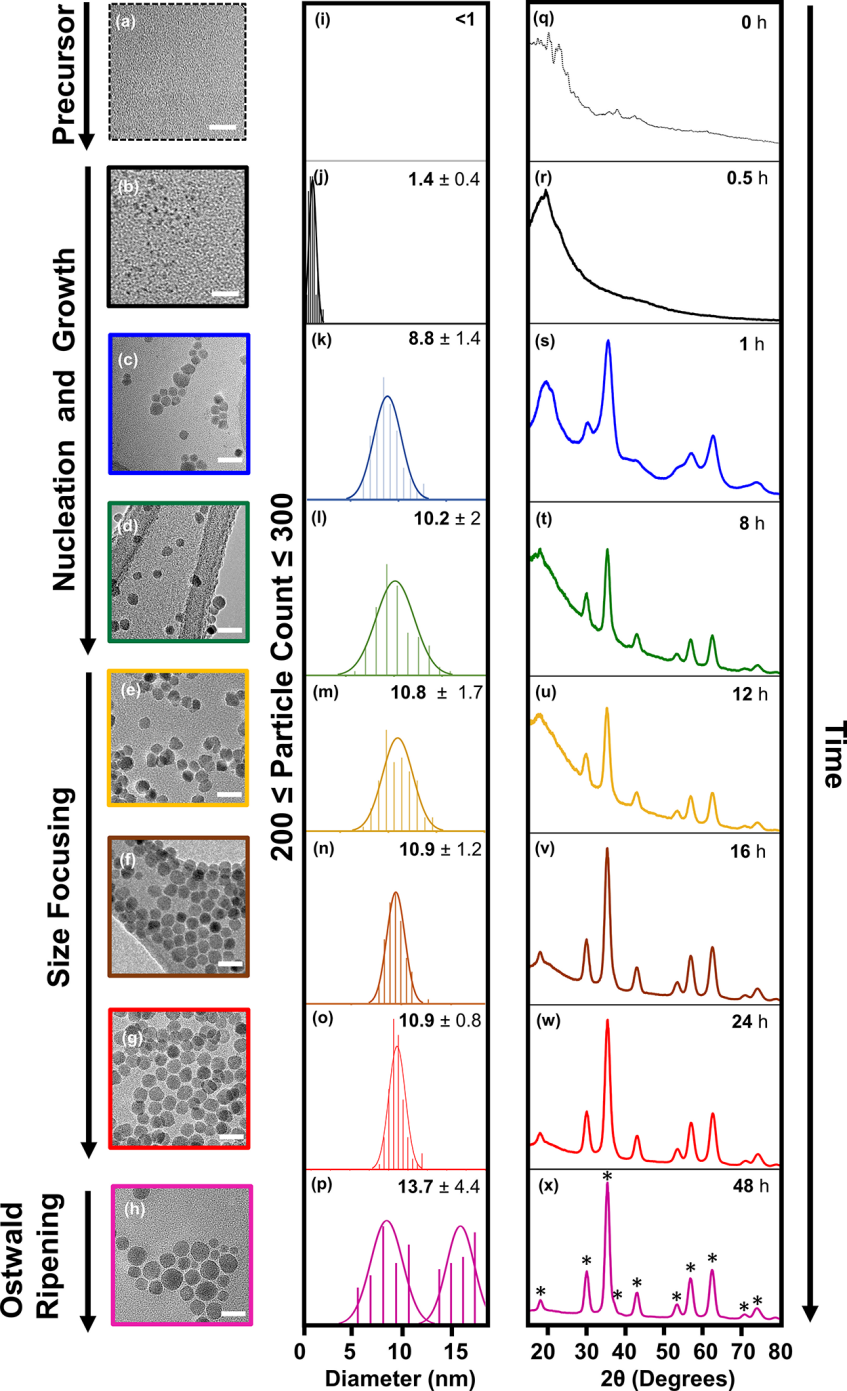
(a–h) Representative TEM images of ZnFe_2_O_4_ nanocrystals obtained after various reaction times under solvothermal conditions in the presence of oleic acid and oleylamine in benzyl ether. Each scale bar represents 20 nm. (i–p) Histograms tabulating nanocrystal diameters obtained after performing particle size measurements/analysis of corresponding TEM micrographs in ImageJ.^42^ Each histogram contains measurements of at least 200 different particles. (q–x) Powder XRD diffractograms of the corresponding products obtained after various reaction times. The * symbol indicates the position of peaks associated with the cubic spinel phase.

To investigate the mechanism by which ZnFe_2_O_4_ nanocrystals form from precursor **1** under solvothermal reaction conditions, we varied the amount of time autoclave reactors spent at the reaction temperature of 230 °C. Reaction temperatures below 200 °C produced nanoparticles with poor crystallinity (see the Supporting Information). Figure 2 displays representative transmission electron microscopy (TEM) images and powder X-ray diffraction (XRD) spectra that illustrate the impact of reaction time on the shape, size, and crystallinity of ZnFe_2_O_4_ nanoparticles. TEM micrographs (Figure 2a–h), resulting size histograms (Figure 2i–p), and corresponding powder XRD spectra (Figure 2q–x) demonstrate that ZnFe_2_O_4_ nanoparticles evolve from small amorphous particles to uniform isotropic nanocrystals over the course of 12 h. After 30 min of reaction time, we observe the formation of small amorphous nuclei with a mean diameter of 1.4 ± 0.4 nm. The possibility of the observed nuclei to be **1** was dismissed after attempting to collect TEM images of this cluster molecule, which we found was not discernible at the resolution of this electron microscopy tool (Figure 2a). After 1 h of reaction time, we recover spherical nanocrystals with an average diameter of 8.8 ± 1.4 nm and the spinel crystal structure. In contrast to previous reports of the synthesis of MFe_2_O_4_ nanocrystals via hot injection of MFe_2_O(oleate)_6_ precursors,^45,46^ we do not observe nucleation of binary iron oxide phases at early reaction times; rather, the only crystalline phase we observe throughout the reaction is the spinel phase. Increasing the reaction time to 8 h results in additional growth to a diameter of 10.2 ± 2 nm and improved crystallinity as evidenced by the sharpening of the peaks in the powder XRD spectrum. For reaction times longer than 8 h, we observe size focusing rather than additional particle growth (Figure 2l-p and Figure S5 in the Supporting Information). At *t* = 24 h, ZnFe_2_O_4_ NCs with a narrow size distribution and an average size of 10.9 ± 0.8 nm are achieved. Increasing the reaction time further, to 48 h, produces a bimodal size distribution that is indicative of Ostwald ripening.^51,52^

Selected area electron diffraction (SAED) patterns of nanocrystals obtained after 24 h of reaction time contain characteristic diffraction rings that are consistent with the pXRD pattern of cubic spinel zinc ferrite (see Figure S6in the Supporting Information). The formation of well-dispersed and stoichiometric ZnFe_2_O_4_ NCs was further confirmed by high-angle annular dark-field scanning transmission electron microscopy (HAADF-STEM) and energy-dispersive X-ray spectroscopy (EDS) mapping images (see the Supporting Information).

**Figure 3:**
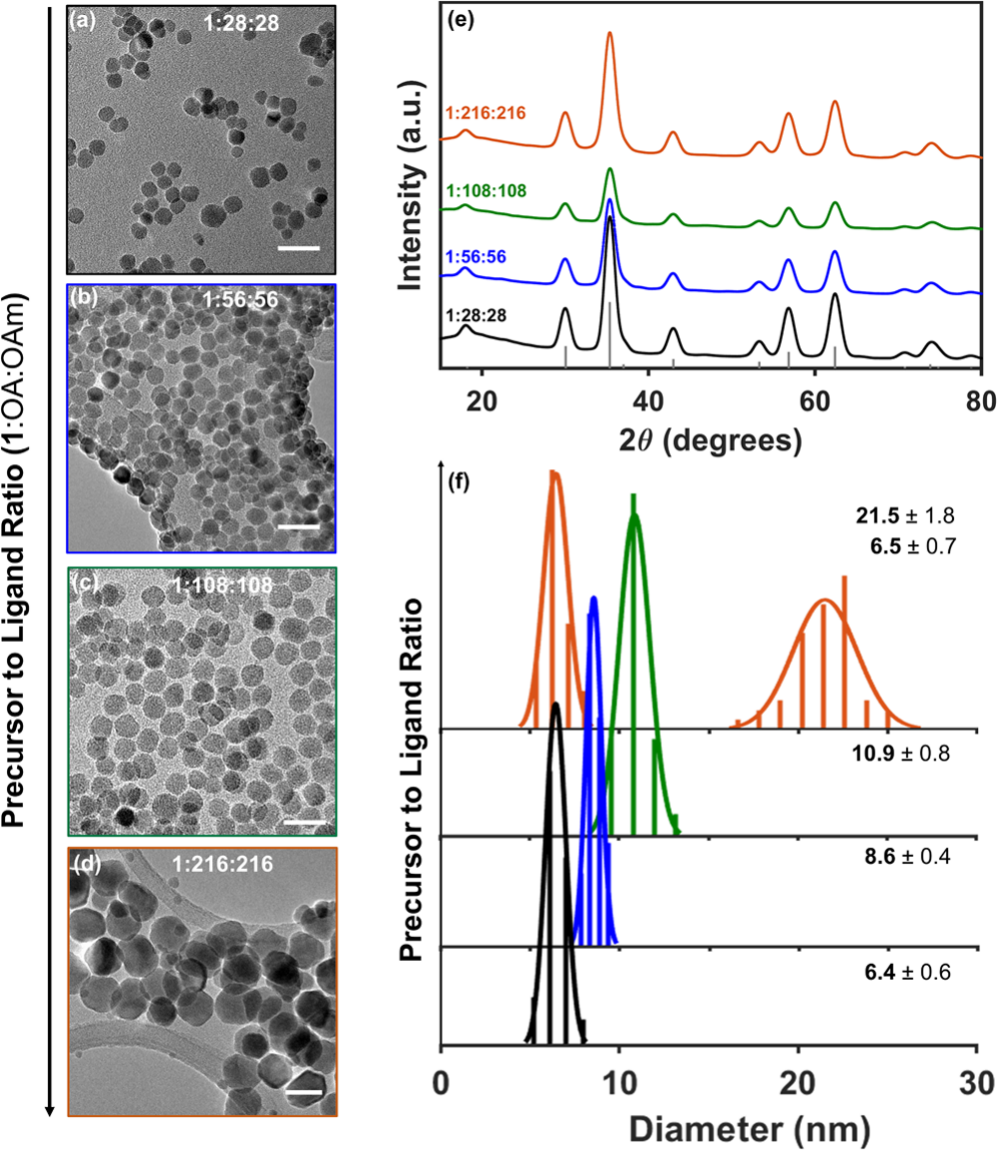
(a–d) Representative TEM images of ZnFe_2_O_4_ NCs obtained from reactions containing different molar ratios of oleic acid and oleylamine to single-source precursor complex **1**. Scale bars correspond to 20 nm. (e) Powder XRD data demonstrating retention of the spinel cubic phase across all precursor to ligand ratios. (f) Plot of size distributions highlighting the formation of a bimodal size distribution at a precursor to ligand ratio of 1:216:216. The labels correspond to the average and standard deviation of the particle diameter each peak in the histograms and are reported in units of nm.

Previous work from our group demonstrates that carboxylic acid and amine ligands impact the size and morphology of hematite (α-Fe_2_O_3_) nanocrystals synthesized via solvothermal reaction of iron(III) chloride in a polar reaction medium comprising water and ethanol.^50^ To examine whether these ligands play a similar role in the solvothermal reaction of heterobimetallic single-source precursors in an organic solvent, we investigated the impact of changing the concentrations of oleic acid and oleylamine on the resulting ZnFe_2_O_4_ nanocrystals. We conducted four reactions to vary the molar ratio between the single-source precursor and the ligands (Table S3). In Figure 3a–c, we observe that increasing the amount of oleic acid and oleylamine relative to the cluster precursor from **1**:OA:OAm = 1:28:28 to 1:108:108 produced spherical ZnFe_2_O_4_ nanocrystals with diameters that increase from 6.4 to 10.9 nm. Increasing the ligand to precursor ratio further (to 1:216:216) produces ZnFe_2_O_4_ nanocrystals with a bimodal size distribution and a mixture of spherical and octahedral morphologies (Figure 3d). Powder XRD confirms that each of these products has a phase-pure spinel crystal structure (Figure 3e). Additionally, energy-dispersive X-ray (EDS) spectra collected in a scanning electron microscope (SEM) confirm that each of these products also contains a stoichiometric 2:1 ratio of iron to zinc (see Figure S8 and Table S5), indicating that changing the ligand ratio does not impact the composition of the final product.

**Figure 4:**
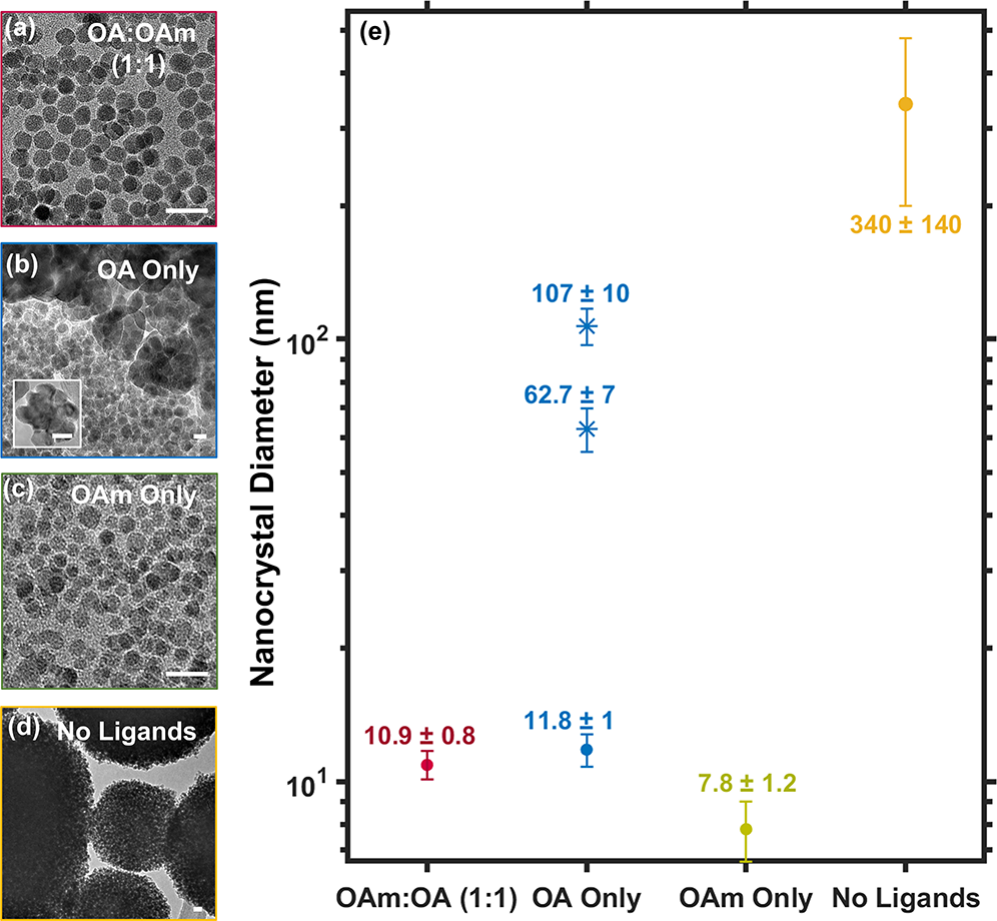
(a–d) Representative TEM images of ZnFe_2_O_4_ nanocrystals synthesized in benzyl ether and (a) the presence of both OA and OAm in a 1:1 ratio, (b) the presence of OA and absence of OAm, (c) the presence of OAm and absence of OA, and (d) the absence of OA and OAm. The scale bars represent 20 nm. (e) Plot of average nanocrystal diameters obtained from the reaction conditions used in parts (a–d).

After establishing that the overall concentration of ligands present in the reaction impacts nanocrystal size, we sought to investigate whether oleic acid (OA) and oleylamine (OAm) impact the reaction in different ways, as has been observed in previously reported syntheses of metal oxide nanocrystals.^19^Figure 4a–d shows representative TEM micrographs of ZnFe_2_O_4_ nanocrystals synthesized in the presence and the absence of oleic acid and/or oleylamine in various combinations, with a precursor-to-ligand ratio fixed at 1:108. Table S6 summarizes the reaction conditions used for this set of experiments and powder X-ray diffraction spectra shown in Figure S9 demonstrate that every combination of ligands used in this set of experiments produces phase-pure spinel nanocrystals (see Supporting Information). EDS data also indicate a 2:1 molar ratio of iron to zinc for each nanocrystal sample shown in Figure 4, again confirming the formation of stoichiometric ternary ZnFe_2_O_4_ nanocrystals (Figure S10 and Table S7 in the Supporting Information). Figure 4a depicts a representative TEM image of ZnFe_2_O_4_ nanocrystals obtained when both ligands, OAm and OA, are present in the reaction system in a 1:1 ratio. These reaction conditions reproducibly produce isotropic and monodisperse nanocrystals with an average size of 10.9 ± 0.8 nm (see the Supporting Information). Figure 4b shows representative TEM images of ZnFe_2_O_4_ nanocrystals obtained in the presence of oleic acid and absence of oleylamine. These nanoparticles tend to form polycrystalline flower-shaped aggregates (see Figure 4b and the Supporting Information). These aggregates account for the trimodal size distribution indicated in Figure 4e, which tabulates the diameters of both single and polycrystalline structures observed in the TEM. Figure 4c shows a representative TEM image of the ZnFe_2_O_4_ nanocrystals obtained from a reaction in which OAm is the only ligand present in the reaction mixture. This reaction produces isotropic nanocrystals with a diameter of 7.8 ± 1.2 nm, which are smaller than those obtained from reactions containing both oleic acid and oleylamine. Figure 4d shows a representative TEM image of the ZnFe_2_O_4_ nanoparticles obtained from a reaction mixture that contained only cluster **1** and benzyl ether, with no additional surfactant ligand. This reaction produces large, spherical polycrystalline agglomerates of phase-pure ZnFe_2_O_4_; the average diameter of these agglomerates is 350 ± 140 nm.

We also explored the effect of using carboxylic acid and amine ligands of various carbon chain lengths on the features of ZnFe_2_O_4_ NCs. Figure S12 in the Supporting Information displays the results from reactions that utilize ligands with three different carbon chain lengths: 18, 12, and 6. Ligands with longer carbon chain lengths (*C* = 18) produced smaller particles with a narrower size distribution (10.9 ± 0.8 nm), while those with shorter carbon chain lengths (*C* = 6) yielded larger particles with broader size distributions (20.5 ± 4 nm) and decreased colloidal stability. These results are similar to what has been previously reported for other metal oxide syntheses.^50^

### Solvent Impacts Morphology, Crystal Phase, and Cation Distribution

One major advantage of the solvothermal approach is that it enables the use of solvents that have boiling points lower than the reaction temperature. The physicochemical properties of the solvent can influence solvothermal processes in several ways. The solvent can induce preferential growth from a specific crystal plane, whereby the organic solvent and/or organic species formed during the reaction act as capping agents and hence control the final morphology of the nanocrystals.^53−55^ The solvent also impacts the solubility of the monomers and thereby the rates of nucleation and growth.^56,57^ Finally, the solvent can act as a reagent and react with the precursors via hydrolysis, alcoholysis, or redox reactions.^49,58^

**Figure 5:**
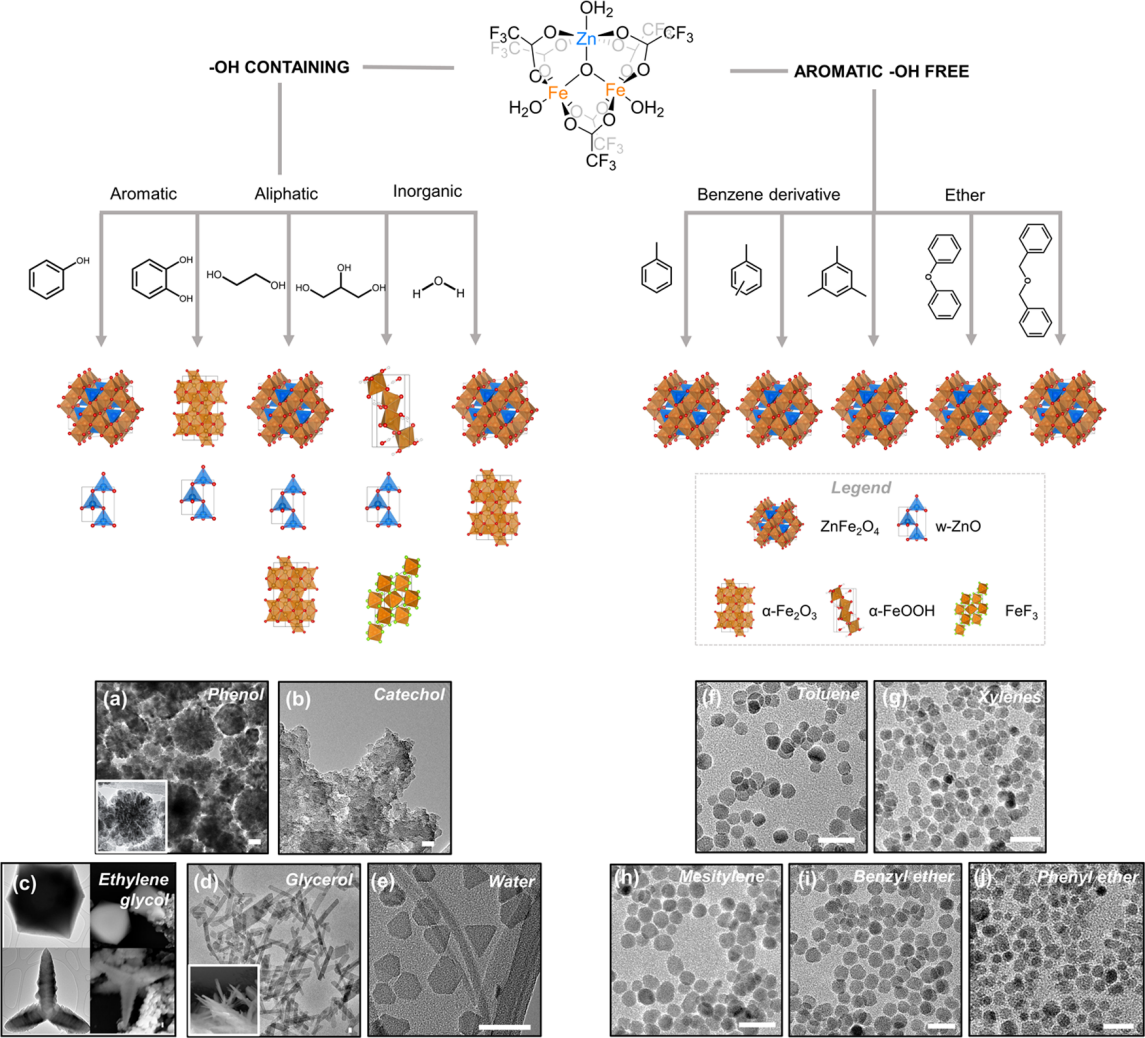
Top: Schematic illustration of the various crystal phases obtained from reacting the single-source precursor **1** with oleic acid and oleylamine in a series of solvents, classified as −OH containing (left) or aromatic −OH-free (right). Bottom: (a–j) Representative TEM images of nanocrystal products obtained from various solvents. The scale bars represent 20 nm.

Here, we observe that the phase and morphology of metal oxide nanocrystals synthesized from our single-source precursor strongly depend on the chemical structure of the solvents used in the solvothermal reaction. Aromatic, aliphatic, and inorganic solvents that contain hydroxyl groups (−OH) not only promote the formation of nanoparticle structures that are diverse in size and shape but also induce the formation of mixtures of different crystal phases. As shown in Figure 5 and summarized in Table 1, we obtain a mixture of ZnFe_2_O_4_, wurtzite ZnO (w-ZnO), and/or α-Fe_2_O_3_ (hematite), when phenol, ethylene glycol, or water is used as the reaction solvent. Specifically, a mixture of w-ZnO and ZnFe_2_O_4_ nanocrystals with flower-like morphologies (average diameter ∼ 56 nm) is obtained from reactions run in phenol. When ethylene glycol is used as the solvent, spheres, tetrapods, and octahedra are formed and we observe peaks corresponding to ZnFe_2_O_4_, w-ZnO, and α-Fe_2_O_3_ in the pXRD spectrum of this product. Reactions run with water as the solvent produce a mixture of α-Fe_2_O_3_ and ZnFe_2_O_4_ nanocrystals with shapes that range from spherical and hexagonal to trigonal but present similar average diameters (∼11 nm). Complete phase segregation into binary phases is observed when catechol or glycerol is used as the reaction solvent. Goethite (α-FeOOH) and even iron fluoride (FeF_3_) (fluoride presumably originating from the precursor trifluoroacetate ligands) are among the resulting crystal phases. XRD spectra of each of these reactions can be found in the Supporting Information. The correlation of specific crystal phases to particular morphologies is beyond the scope of this paper. We hypothesize that the formation of binary phases in the presence of solvents containing hydroxyl groups is driven by the disintegration of the single-source precursor core by solvent molecules via hydrolysis or alcoholysis reactions prior to nanocrystal nucleation. Conversely, this hypothesis implies that the μ_3_-oxo-bridged ZnFe_2_O core of the cluster precursor, **1**, remains intact in reactions that produce phase-pure ZnFe_2_O_4_ nanocrystals.

**Table 1:** Summary of Reaction Conditions and Results^a^

micrograph number	solvent	crystal phase^b^	predominant morphologies (>80%)	size distribution (nm)^c^
5a	phenol	ZnFe_2_O_4_	flower-shape	56 ± 16
		w-ZnO		
5b	catechol	α-Fe_2_O_3_	hedge-hog shaped	70 ± 8
		w-ZnO		
5c	ethylene glycol	ZnFe_2_O_4_α-Fe_2_O_3_	octahedrons	1400 ± 110
		w-ZnO	spheres	7.9 ± 1.0
			tetrapods	1350 ± 160*
5d	glycerol	α-FeOOH	platelets	360 ± 40
		w-ZnO		
		FeF_3_		
5e	water	ZnFe_2_O_4_α-Fe_2_O_3_	polyhedrons	11.2 ± 1.8
5f	toluene	ZnFe_2_O_4_	spheres	13.7 ± 1.3
5g	xylenes	ZnFe_2_O_4_	spheres	11.3 ± 1.0
5h	mesitylene	ZnFe_2_O_4_	spheres	14.8 ± 1.4
5i	benzyl ether	ZnFe_2_O_4_	spheres	10.8 ± 0.8
5j	phenyl ether	ZnFe_2_O_4_	spheres	8.8 ± 2.0

a Set focusing temperature = 230 °C. Precursor (mmol) = 0.025. Oleic acid and oleylamine (mmol) = 2.7.

b Crystal phase code: ZnFe_2_O_4_—zinc ferrite; α-Fe_2_O—hematite; w-ZnO—zinc oxide (wurtzite); FeOOH—goethite; FeF_3_—ferric fluoride.

c Mean particle size calculated from a minimum count of 100 nanoparticles (*n* = 3, mean ± SD). *Average length of pods.

In contrast, aromatic hydroxyl-free solvents, namely, toluene, xylenes, mesitylene, benzyl ether, and phenyl ether, form phase-pure ZnFe_2_O_4_ nanocrystals (Figure 5, see the Supporting Information for XRD data). Transmission electron microscopy reveals that each of these solvents produces spherical nanocrystals but with slightly different diameters (Figure 5f–i, and Table 1). Phenyl ether produces the smallest nanocrystals (*d* = 8.8 ± 2.0 nm) and mesitylene produces the largest (*d* = 14.8 ± 1.4 nm). These results demonstrate that the particle size depends on the solvent media under comparable reaction conditions. Importantly, the exclusion of hydroxyl groups from the solvent system is necessary to achieve phase-pure ternary zinc ferrite nanocrystals.

Although the nanocrystals obtained from reactions run in OH-free aromatic solvents are all phase-pure spinel ZnFe_2_O_4_, they do exhibit some subtle differences in their crystal structures. Table 2 tabulates the lattice parameters of each of these samples, which were determined from the positions of the seven most intense peaks in the pXRD spectrum (see the Supporting Information). The lattice parameter of ZnFe_2_O_4_ is correlated to the cation distribution, with increasing inversion causing a decrease in the lattice parameter due to the contraction of M–O bonds in tetrahedral sites upon exchange of Zn^2+^ for Fe^3+^.^59,60^ To confirm that the variation in the lattice parameter arises from a change in cation distribution, we characterized each sample of ZnFe_2_O_4_ using X-ray photoelectron spectroscopy (XPS).

**Table 2:** Structural Parameters of ZnFe_2_O_4_ Nanocrystals Synthesized in Aromatic Solvents

solvent	lattice parameter (Å)	size (nm)	degree of inversion^a^
xylenes	8.4209	11.3 ± 1.0	0.15 ± 0.05
mesitylene	8.4204	14.8 ± 1.4	0.21 ± 0.07
phenyl ether	8.4156	8.8 ± 2.0	0.22 ± 0.02
toluene	8.4095	13.7 ± 1.3	0.44 ± 0.15
benzyl ether	8.4066	10.8 ± 0.8	0.67 ± 0.001

a Mean degree of inversion obtained from XPS measurements (*n* = 3, mean ± SD).

**Figure 6:**
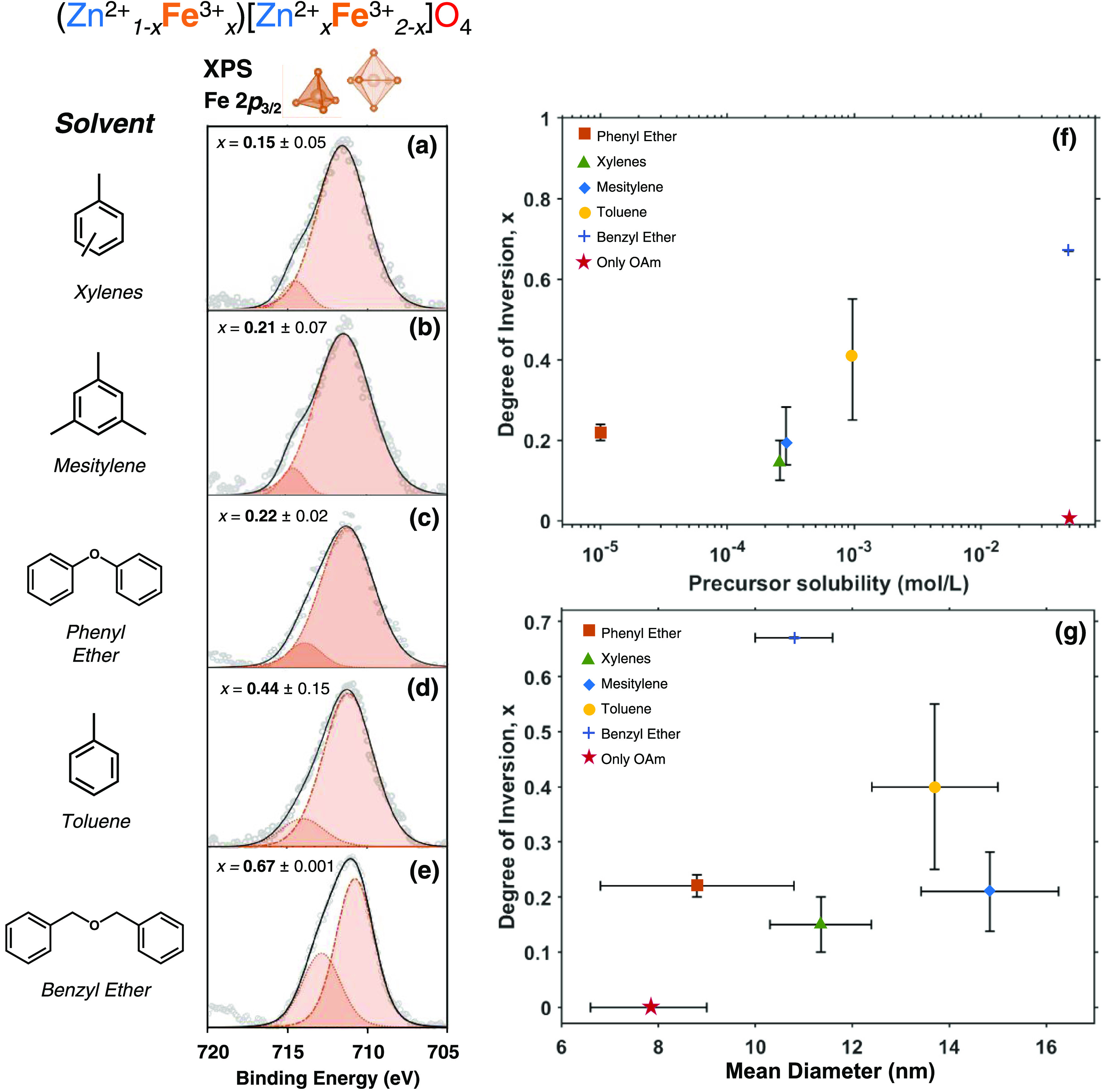
(a–e) X-ray photoelectron spectra of the Fe 2p peak collected from dropcast films of ZnFe_2_O_4_ nanocrystals synthesized in xylenes (a), mesitylene (b), phenyl ether (c), toluene (d), and benzyl ether (e). The orange shading depicts Gaussian peak fits to these data. Plots of the inversion parameter *x* versus precursor solubility (f) and nanocrystal diameter (g). The vertical error bars depict the uncertainty in *x* determined from the standard deviation of three different measurements obtained from three different locations on the same sample. The horizontal error bars in (g) depict the standard deviation of the nanocrystal size distribution. The pink star in (f) and (g) refers to the reaction system with only oleylamine as the surfactant ligand and benzyl ether as the solvent.

We analyzed the cation distribution of Fe^3+^ across tetrahedral and octahedral sites by collecting XPS data within the Fe 2p region.^61−64^ Fe^3+^ ions occupying tetrahedral sites exhibit larger binding energies for 2p electrons than Fe^3+^ ions occupying octahedral sites because they are coordinated to fewer O^2–^ anions and therefore have less electron density in their coordination sphere.^65^Figure 6 shows X-ray photoelectron spectra collected of the Fe 2p_3/2_ peak for ZnFe_2_O_4_ nanocrystals synthesized in various aromatic solvents. Each of these peaks exhibits an asymmetry that indicates that the inversion parameter is greater than zero. We fit these peaks to two Gaussian components: the higher-energy component centered at 713.0 eV is assigned to Fe^3+^ ions occupying tetrahedral sites and the lower-energy component centered at 710.7 eV is assigned to Fe^3+^ occupying octahedral sites. The fraction of the total peak area that is occupied by the tetrahedral Fe^3+^ peak corresponds to the inversion parameter, *x*, via eq 1, where is the area under the higher-energy peak and  is the area under the lower-energy peak.
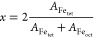
1Table 2 lists the values of *x* obtained for each nanocrystal sample synthesized in an OH-free aromatic solvent. Consistent with bulk materials, *x* is inversely related to the lattice parameter: as *x* increases, the lattice parameter decreases (see the Supporting Information).

Figure 6g plots the inversion parameter versus the average size of the nanocrystals. This plot demonstrates that the values of *x* that we measure do not correlate with the size or surface area-to-volume ratio of the nanocrystals, which indicates that the contribution of under-coordinated surface cations to the measured value of *x* is minimal. Instead, the inversion parameter exhibits a positive correlation with the solubility of precursor complex **1** in each of the reaction solvents, as shown in Figure 6f. The procedure used to determine the solubility of **1** in these solvents is described in the Supporting Information. Other properties of the solvents, such as density, dielectric constant, and boiling point, exhibit no correlation with the inversion parameter of the ZnFe_2_O_4_ nanocrystals (see the Supporting Information).

## Discussion

**Scheme 1:**
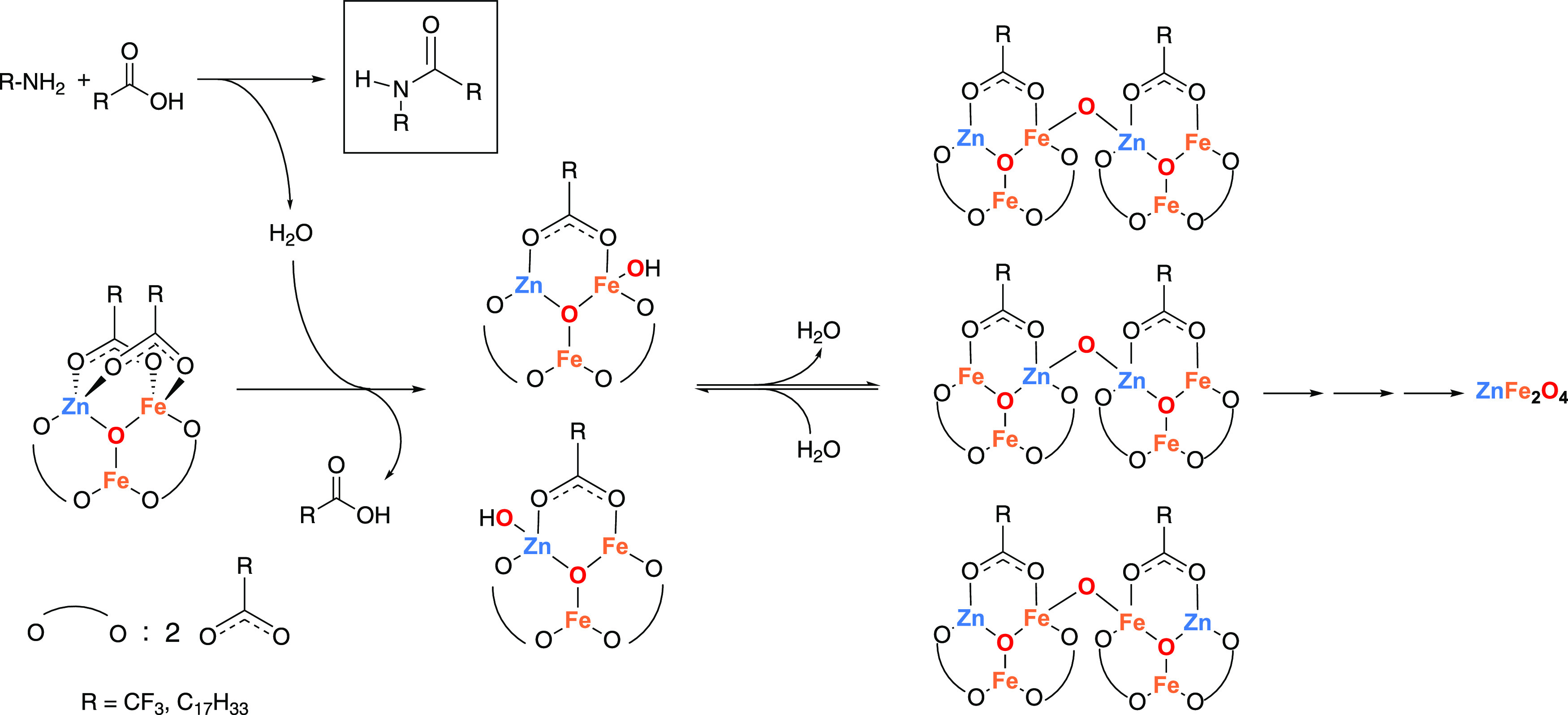
Proposed Mechanism for Formation of ZnFe_2_O_4_ from Single-Source Precursor **1**

In our previous work, we hypothesized that the mechanism of nanocrystal formation from the solvothermal reaction of trinuclear, carboxylate-bridged single-source precursor complexes, such as **1**, in the presence of oleic acid and oleylamine, proceeds via nucleophilic attack of a bridging carboxylate by oleylamine to form an amide byproduct (detected by FTIR of the reaction supernatants) and a cluster containing a hydroxylated metal center.^18^ Condensation of two hydroxylated clusters to form a new bridging metal–oxo–metal moiety initiates nucleation of a ZnFe_2_O_4_ nanocrystal (Scheme 1). Based on the results presented in the previous sections, we propose one additional pathway for precursor conversion to the hydroxylated cluster: direct hydrolysis upon reaction with water that originates from the coordinated water molecules in the cluster precursor complex **1** or is formed upon condensation of the free carboxylic acid with the free amine to form the amide byproduct (Scheme 1). In the next two sections, we discuss how this proposed mechanism explains the observed impacts of carboxylate and amine ligands and reaction solvent on the size and cation distribution of ZnFe_2_O_4_ nanocrystals.

### Impacts of Ligands and Solvent on the Size of ZnFe_2_O_4_ Nanocrystals

The results in Figure 4e indicate that the presence of oleylamine alone is sufficient to prevent aggregation and promote formation of monodisperse nanocrystals. Oleylamine in particular, along with primary amine ligands in general, has a similar impact on many other nanocrystal synthesis reactions including solvothermal synthesis of metal oxide nanocrystals and ambient pressure heat-up or hot-injection syntheses of metal chalcogenide and noble metal nanoparticles.^50,66−69^ The dative donation of the electron lone pair on the nitrogen to atoms on the surfaces of growing nanocrystals provides a moderate surface binding affinity that results in a dynamic population of surface-bound amines that, on average, is dense enough to protect the surface and prevent aggregation but not so dense that it prevents nanocrystal growth. Addition of oleic acid promotes formation of larger nanocrystals but also leads to aggregation in the absence of oleylamine. Increased concentration of oleic acid and oleylamine also promotes formation of larger nanocrystals (Figure 3).

We propose two possible mechanisms whereby addition of oleic acid (or other carboxylic acids) leads to formation of larger nanocrystals. In our first proposed mechanism, the carboxylic acid reacts with the amine to form an amide bond and evolve water. Water promotes the hydrolysis reactions that drive precursor conversion and therefore increases the rate at which reactive monomers become available. Water can also increase the rate of growth by hydrolyzing the surface of the growing nanocrystal to produce reactive surface hydroxyl sites that can undergo condensation with incoming monomer species. The net effect of these increased rates is the formation of larger nanocrystals. Our second proposed mechanism posits that the addition of carboxylic acid increases the availability of accessible protons. Increased proton concentration can also increase the rates of hydrolysis reactions^70^ and thereby lead to larger nanocrystals as discussed above. Although we cannot rule out either of these mechanisms, given the largely nonpolar nature of the reaction solvent, we suspect that the first mechanism involving evolution of water is dominant. We note that both of these mechanisms are consistent with our observation that the average size of the nanocrystals increases when the concentrations of both oleic acid and oleylamine are increased together (Figure 3a–d).

In addition to reacting with amines, carboxylic acid ligands can also displace the trifluoroacetate ligands on the precursor molecule **1** and bind to the surface of the growing nanocrystals. The Supporting Information contains ^19^F NMR data demonstrating the displacement of trifluoroacetic acid from cluster **1** upon addition of oleic acid at room temperature. This ligand exchange reactivity combined with our observation that carboxylic acid ligands with shorter carbon chain lengths produce larger nanocrystals suggests that diffusion of various species in solution, including ligand-exchanged precursor molecules, monomers, ligands, nuclei, and the growing nanocrystals, impacts the final nanocrystal size. Presumably, smaller ligands result in faster diffusion and faster reaction rates. It is also possible that the smaller steric barriers presented by the shorter chain ligands improve the ability of water to hydrolyze the cluster precursor and the ability of monomers to access the nanocrystal surfaces, thereby promoting faster nucleation and growth.

Finally, carboxylate ligands are known to etch metal ions from the surfaces of metal oxides,^71,72^ which can lead to surface destabilization and aggregation. We suspect that such processes may be responsible for the increased aggregation observed in the presence of oleic acid and the absence of oleylamine.

### Impacts of Solvent and Ligands on Cation Distribution in ZnFe_2_O_4_ Nanocrystals

For bulk ZnFe_2_O_4_, the most thermodynamically stable value for the degree of inversion is 0 for two reasons: (i) placing the more charged Fe^3+^ ions in the octahedral sites where they are surrounded by a greater number of anions provides greater coulombic stabilization than placing them in the tetrahedral sites and (ii) mixing of the valence 4s and 4p orbitals on Zn^2+^ favors a tetrahedral geometry that enables sp^3^-type bonding interactions with the coordination sphere.^27,30^ Therefore, our observation of inversion parameters larger than 0 indicates that our reactions are driven, at least in part, by kinetic control. Notably, the largest value of *x* that we observe is 0.67, which corresponds to a fully randomized distribution of Zn^2+^ and Fe^3+^ ions among the tetrahedral and octahedral sites. This maximum value further supports the notion that increased kinetic rather than thermodynamic control drives changes in *x* for reactions run in different solvents. We therefore hypothesize that modulation of kinetic barriers in precursor conversion, nucleation, or growth steps enables tuning of the inversion parameter.

**Figure 7:**
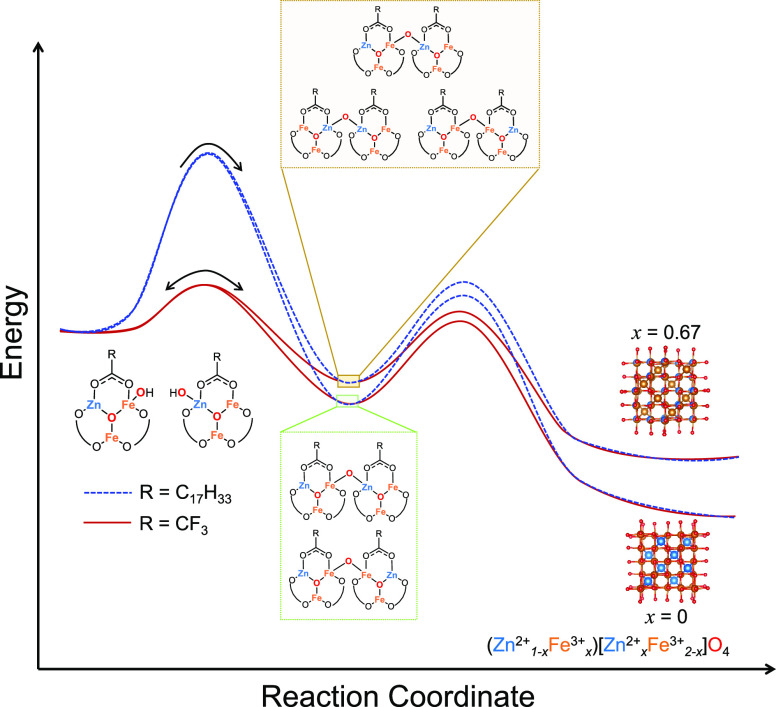
Conceptual illustration of proposed reaction coordinate diagrams for conversion of hydroxylated precursor molecules to ZnFe_2_O_4_ nanocrystals with various cation distributions. The solid red lines depict the reaction pathways available to the precursors that retain trifluoroacetate ligands and the dashed blue lines depict reaction pathways available to precursors that have undergone ligand exchange with oleic acid.

The condensation reaction that couples two hydroxylated precursor clusters together to form new metal–oxygen bonds and initiate nucleation produces three possible configurations of M–O–M moieties: Zn–O–Fe, Fe–O–Fe, and Zn–O–Zn. The cation distribution of the resulting nanocrystal depends on the relative populations of the various pairwise combinations of M–O–M bonds, with Zn–O–Zn linkages required to achieve inversion (*x* > 0). Given the assumption that there is no significant preference for any particular metal center to be hydroxylated (see the Supporting Information for justification of this assumption), the initial distribution of different types of M–O–M linkages should be purely statistical. The ability to access a distribution of M–O–M linkages that leads to the more thermodynamically favorable cation distributions (i.e., a minimal number of Zn–O–Zn linkages leading to minimal inversion) depends on the system’s ability to sample many different combinations based on the microscopic reversibility of the condensation reaction. This reversibility in turn depends on the height of the kinetic barrier to condensation, which, we argue, depends at least in part on the structure of the carboxylate ligands bound to the cluster molecules. Bulky ligands, like oleic acid, present steric barriers that limit access to the M–O–M linkages and thereby limit the hydrolysis (i.e., reverse condensation) reactions. Thus, these ligands are more likely to “lock in” a random, statistical distribution of M–O–M linkages that leads to a random cation distribution than smaller ligands like trifluoroacetic acid. Figure 7 depicts a conceptual sketch of the reaction coordinate diagram for conversion of hydroxylated cluster precursors to nanocrystals that illustrates the proposed impact of oleic acid on the activation barrier to the initial condensation of these molecules.

We tested our hypothesis that ligand-mediated kinetic control of nanocrystal formation promotes cation inversion by investigating the impact of reaction temperature and oleic acid concentration on the cation distribution of ZnFe_2_O_4_ nanocrystals (see the Supporting Information). Decreasing the temperature of the reaction conducted in xylenes from 230 to 200 °C resulted in an increase in the inversion parameter from 0.15 ± 0.5 to 0.35 ± 0.02. This result is consistent with our hypothesis that there is a kinetic barrier to achieving a small inversion parameter. Removing oleic acid from the reaction of **1** in benzyl ether at 230 °C produced nanocrystals with an inversion parameter of *x* = 0, which represents the thermodynamically stable product and is much smaller than the inversion parameter obtained in the presence of oleic acid (*x* = 0.67) (Figure 6f). This result indicates that the presence of oleic acid increases the height of the kinetic barrier associated with minimizing inversion. Decreasing the temperature of the oleic acid-free reaction in benzyl ether from 230 to 200 °C increases the inversion parameter from 0 to 0.69 ± 0.09, further supporting the presence of this kinetic barrier. Our observation of a kinetically controlled cation distribution obtained from a reaction that is run for 24 h combined with our observation that the nanocrystals achieve their final average size only 12 h into the reaction indicates that cation rearrangement to a more thermodynamically stable distribution within an already formed nanocrystal does not occur under these reaction conditions. Thus, we propose that the kinetic barrier to achieving a thermodynamic cation distribution is operative at the nucleation step that involves condensation of two hydroxylated clusters and at growth steps involving addition of a hydroxylated cluster monomer to the surface of a nanocrystal via condensation. Importantly, this method for controlling the cation distribution requires the reactive monomers to contain intact μ_3_-oxo-bridged Zn–O–Fe_2_ units that originate from the cluster precursor. We attempted to test this hypothesis by characterizing the cation distribution of ZnFe_2_O_4_ nanocrystals synthesized from a mixture of mononuclear multi-source precursors, namely, zinc(II) nitrate and iron(III) nitrate; however, XPS analysis of the nanocrystalline spinel oxide products of this reaction indicates the presence of both Fe^3+^ and Fe^2+^ species (see the Supporting Information). Although the binding energy of the Fe^3+^ peak is most consistent with octahedral Fe^3+^ sites, the presence of mixed valent iron centers makes it difficult to accurately quantify the inversion parameter using XPS.

Our observations that hydrolyzing solvents produce binary oxide side products and nonhydrolyzing solvents exclusively nucleate the ternary spinel ZnFe_2_O_4_ phase also indicate that the Zn–O–Fe_2_ units of the cluster structure remain intact in the reactive monomers. This model enables us to explain our observation that the value of *x* measured for ZnFe_2_O_4_ nanocrystals synthesized in different solvents increases with the solubility of **1** in each solvent (Figure 6f). We hypothesize that the solubility of a cluster precursor dictates its availability for the ligand exchange reaction. More soluble clusters have more contact with oleic acid and therefore are more likely to undergo ligand exchange with oleic acid prior to hydrolysis/condensation. These ligand exchange reactions facilitate kinetic trapping of inverted cation distributions and thus produce nanocrystals with larger values of *x*.

## Conclusions

The results presented here demonstrate that the concentration and chemical structure of ligands and solvent present during the solvothermal synthesis of ZnFe_2_O_4_ nanocrystals from a single-source precursor influence the size, monodispersity, and cation distribution of the nanocrystals in ways that are both similar to and distinct from what has generally been observed for other colloidal nanocrystal systems. As in other nanocrystal syntheses, both those conducted under solvothermal conditions and at ambient pressure, the presence of oleylamine suppresses aggregation and improves monodispersity, while excess carboxylic acid leads to aggregation. Here, we observe that increasing the overall concentration of both oleic acid and oleylamine by the same amount leads to larger nanocrystals, which we attribute to an increased concentration of water generated upon reaction of oleic acid and oleylamine to form an amide. The ability to tune the cation distribution by tuning the extent of ligand exchange between oleic acid and the carboxylate-bridged cluster precursors is unique to the synthetic method presented here and is enabled by two key features. First, the solvothermal reaction conditions enable the use of solvents at reaction temperatures that exceed their boiling points and thus provide a facile way to tune the efficiency of ligand exchange without requiring the synthesis of a large library of precursor clusters or using other carboxylate ligands that may compromise the colloidal stability of the resulting nanocrystals. Second, the use of a single-source precursor containing an oxo-bridged Zn–O–Fe_2_ core that remains intact through precursor conversion, nucleation, and growth provides a platform by which ligand exchange can influence the assembly of Zn–O–Fe_2_ units to form a nanocrystal.

This work represents the first demonstration of rational independent synthetic control over both the cation distribution and size of monodisperse colloidal ternary ZnFe_2_O_4_ nanocrystals, and this approach should be applicable to other ternary spinel ferrite compositions. Such synthetic control will enable systematic investigations into the influence of cation distribution on the performance of these nanocrystals in various applications, such as photocatalysis and magnetic imaging.

## Supporting Information Available

Details of hot-injection and heat-up procedures; additional characterization of ZnFe_2_O_4_ nanocrystals synthesized under various reaction conditions (ligand concentration and chain length, temperature, solvent, multisource precursors, etc.) via EDS, TEM, XPS, and powder X-ray diffraction; description of the crystal structure of **1**; determination of the solubility of **1** in various solvents; lattice constant calculations; and characterization of ligand exchange between **1** and oleic acid via UV–vis absorption, ^1^H NMR, and ^19^F NMR (PDF)
Crystallographic information file for cluster **1** (CIF)

## Supplementary Material

cm2c01568_si_001.pdf

cm2c01568_si_002.cif
