# Supplementary material for: Controlling Cation Distribution and Morphology in Colloidal Zinc Ferrite Nanocrystals
Source: Chem Mater. 2022 Aug 1;34(16):7446–59. doi: 10.1021/acs.chemmater.2c01568 (PMC9417087; doi:10.1021/acs.chemmater.2c01568)
Supplement: Supplementary file 1 — cm2c01568_si_001.pdf [file cm2c01568_si_001.pdf]

Supporting Information for

Controlling Cation Distribution and Morphology in Colloidal Zinc Ferrite Nanocrystals

Karla R. Sanchez-Lievanos and Kathryn E. Knowles*

Department of Chemistry, University of Rochester, Rochester, NY 14627, United States

*Corresponding author: Email address: kknowles@ur.rochester.edu

TABLE OF CONTENTS

Hot Injection Procedure 3

Heat-up Procedure 3

^{1}H and ^{19}F Nuclear Magnetic Resonance (NMR) 3

Conversion of the XRD pattern taken using Molybdenum Kα to Copper Kα 3

Crystallographic Characterization of Cluster Precursor **1** 4

Table S1. Crystal data and structure refinement for **1**. 6

Table S2. Structural comparison of cluster **1** and a previously reported structure with the same composition but different crystallization solvent molecules. 7

Figure S2. FTIR spectra of the cluster **1** and free trifluoroacetic acid (TFA). 7

Figure S3. Representative TEM micrographs of the products obtained from hot injection and heat up methods carried out at a reaction temperature of 230 °C. 7

Figure S4. Powder X-ray diffraction spectra of nanoparticles obtained from solvothermal reactions of cluster precursor **1** at various temperatures. 8

Figure S5. Plot of average diameter of $ZnFe_2O_4$ nanocrystals versus reaction time 8

Figure S6. Selected area electron diffraction (SAED) patterns and STEM elemental mapping of of $ZnFe_2O_4$ nanocrystals 9

Figure S7. Representative TEM images of $ZnFe_2O_4$ nanocrystals obtained from three different reactions run under identical conditions 10

Table S3. Statistical Analysis of the Diameters of $ZnFe_2O_4$ Nanocrystals Synthesized from cluster **1** with a **1**:OA:OAm ratio of 1:108:108 10

Table S4. Summary of reaction conditions used to vary the precursor to ligand ratio and the morphology of the resulting nanocrystals 11

Figure S8. Energy dispersive X-ray spectra of $ZnFe_2O_4$ nanocrystals synthesized using different precursor to surfactant ligands ratios 11

Table S5. Atomic percent composition of Zn and Fe in $ZnFe_2O_4$ NPs synthesized using different precursor to surfactant ligands ratios (**1**:OA:OAm) measured by EDS. 11

Table S6. Summary of reaction conditions used to vary the ligand composition and the morphology of the resulting nanocrystals. 12

Figure S9. Powder X-ray diffraction patterns of reaction products obtained in the presence of different ligand mixtures 12

Figure S10. Energy dispersive X-ray spectra of $ZnFe_2O_4$ nanocrystals synthesized at 230 °C in benzyl ether as solvent containing different ligand mixtures. 13

Table S7. Atomic Percent Composition of Zn and Fe in $ZnFe_2O_4$ NPs Synthesized in the Presence of Different Ligand Mixtures as Measured by EDS 13

Figure S11. Representative TEM images of products obtained after various reaction times 13

Figure S12. Representative TEM images and powder XRD spectra of $ZnFe_2O_4$ nanocrystals obtained from the reaction of **1** in the presence of ligands of decreasing carbon chain lengths. . 14

Figure S13. Powder X-ray diffraction spectra of nanocrystal samples obtained from solvothermal reactions in solvents containing hydroxyl groups. 15

Figure S14. Powder X-ray diffraction spectra of nanocrystals obtained from solvothermal reactions in aromatic solvents that do not contain hydroxyl groups. 15

Table S8. Summary of reaction conditions used for various reaction solvents. 16

Lattice Parameter Calculation 16

Table S9. Powder X-ray diffraction data used to determine the lattice parameter of $ZnFe_2O_4$. ... 17

Figure S15. Plot of lattice constant determined from powder X-ray diffraction data versus the inversion parameter x for $ZnFe_2O_4$ nanocrystals synthesized in aromatic OH-free solvents. 18

Determination of Solubility of **1** in Various Reaction Solvents 19

Figure S16. Absorption spectra and Beer-Lambert's law plot used to determine the molar extinction coefficient of **1** 19

Table S10. Absorbance data used to determine the concentrations of saturated solutions of **1** ... 19

Table S11. Physicochemical properties of solvent media 20

Figure S17. Absorbance and ^{1}H NMR data characterizing the ligand exchange reaction between **1** and oleic acid 20

Characterization of ligand exchange with cluster **1** by ^{19}F NMR 21

Figure S18. ^{19}F NMR spectra characterizing cluster **1** and its ligand exchange with oleic acid .. 22

Figure S19. Plots of the Fe $2p_{3/2}$ region of the x-ray photoelectron spectra of $ZnFe_2O_4$ nanocrystals synthesized in two different reaction mixtures and at two different temperatures .. 23

Figure S20. Representative X-ray photoelectron survey spectra of the $ZnFe_2O_4$ nanocrystals synthesized from cluster **1** 23

Note on the impact 2+ vs 3+ metal charge has on ligand exchange/hydrolysis 24

Figure S21. Characterization of the nanocrystals obtained from multi-source precursors 24

Hot Injection Procedure

In a typical reaction, oleylamine (OAm, 2.7 mmol), oleic acid (OA, 2.7 mmol), and solvent (9 mL) were degassed at 100 °C under vacuum for 30 min and then heated to 230 °C. The precursor **1** (0.025 mmol) was dispersed in benzyl ether (1 mL) and injected into the solution of the hot mixture. The reaction was maintained at 230 °C for 1 h. The dark mixture was allowed to cool, then it was purified with three cycles of precipitation with acetone followed by centrifugation. This procedure was also performed at a reaction temperature of 290 °C.

Heat-up Procedure

In a 25 mL three-neck round-bottom flask equipped with a reflux condenser, precursor **1** (0.025 mmol) was mixed with oleylamine (OAm, 2.7 mmol), oleic acid (OA, 2.7 mmol), and benzyl ether (10 mL) forming a dark red solution. This mixture was degassed by alternating between vacuum and N_2 flow for 3 times at room temperature, followed by heating under vacuum to 110°C for 1.5 h. Afterward, the mixture was heated (25°C/min) to 230°C under N_2. The solution did not change color. This procedure was also performed at a reaction temperature of 290 °C.

^{1}H and ^{19}F Nuclear Magnetic Resonance (NMR)

All ^{19}F NMR experiments were performed at room temperature on a Bruker 400 MHz spectrometer. All ^{1}H NMR spectra were collected on a Varian 500 MHz spectrometer. Chemical shifts are reported in parts per million (ppm).

Conversion of the XRD pattern taken using Molybdenum Kα to Copper Kα

To convert a pattern obtained from diffraction of X-rays generated from Mo Kα emission to the equivalent pattern for diffraction of X-rays generated from Cu Kα emission, the data first needs to be converted into Q-space using equation S1, where λ_{Mo} and θ_{Mo} are the wavelength Bragg angles for diffraction of Mo Kα emission, respectively.

$$Q = \frac{4\pi sin(\theta_{Mo})}{\lambda_{Mo}} \quad \text{(S1)}$$

The Bragg angles for diffraction of the Cu Kα radiation, θ_{Cu}, can then be calculated by rearranging equation S1 and replacing λ_{Mo} = 0.71073 Å with λ_{Cu} = 1.54148 Å to generate equation S2.

$$\theta_{Cu} = arcsin\left(\frac{Q\lambda_{Cu}}{4\pi}\right) \quad \text{(S2)}$$

We note that this conversion is only appropriate for comparing positions of diffraction peaks. Any analysis of line shapes should be carried out using the originally collected data.

Crystallographic Characterization of Cluster Precursor 1

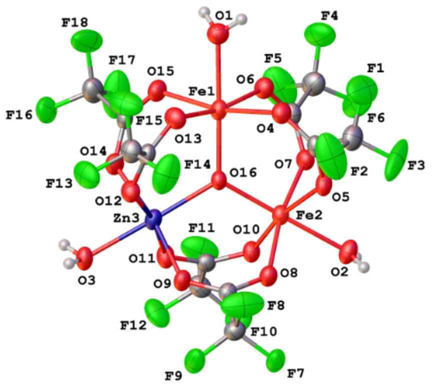

Figure S1. Crystal structure of $ZnFe_2(\mu_3\text{-}O)(\mu_2\text{-}O_2CCF_3)_6(H_2O)_6$ (**1**).

Data collection

A crystal (0.217 x 0.062 x 0.027 mm^3) was placed onto a thin glass optical fiber or a nylon loop and mounted on a Rigaku XtaLAB Synergy-S Dualflex diffractometer equipped with a HyPix-6000HE HPC area detector for data collection at 100.00(10) K. A preliminary set of cell constants and an orientation matrix were calculated from a small sampling of reflections.[1] A short pre-experiment was run, from which an optimal data collection strategy was determined. The full data collection was carried out using a PhotonJet (Cu) X-ray source with frame times of 0.51 and 2.04 seconds and a detector distance of 31.2 mm. Series of frames were collected in 0.50º steps in ω at different 2θ, κ, and ϕ settings. After the intensity data were corrected for absorption, the final cell constants were calculated from the xyz centroids of 32584 strong reflections from the actual data collection after integration.[1] See Table 1 for additional crystal and refinement information.

Structure solution and refinement

The structure was solved using SHELXT[2] and refined using SHELXL.[3] The space group *P*-1 was determined based on intensity statistics. Most or all non-hydrogen atoms were assigned from the solution. Full-matrix least squares / difference Fourier cycles were performed which located any remaining non-hydrogen atoms. All non-hydrogen atoms were refined with anisotropic displacement parameters. Ordered hydrogen atoms that participate in hydrogen bonding were found from the difference Fourier map and refined freely. All other hydrogen atoms were placed in ideal positions and refined as riding atoms with relative isotropic displacement parameters. The

final full matrix least squares refinement converged to $R1 = 0.0471$ (F^2, $I > 2\sigma(I)$) and $wR2 = 0.1230$ (F^2, all data).

Structure description

The structure is shown in Figure S2. The asymmetric unit contains one Fe_2Zn cluster, four cocrystallized acetone solvent molecules, and one cocrystallized water solvent molecule, all in general positions. Each metal site was modeled as a disorder of Fe and Zn: Fe1:Zn1, 0.89:0.11, Fe2:Zn2, 0.77:0.23, Fe3:Zn3, 0.34:0.66. The total metal ratio was constrained to 2Fe:1Zn. CF_3 group C2-F1-F2-F3 is modeled as disordered over two positions (0.58:0.42). One cocrystallized water and two acetone solvent molecules were modeled as disordered over two positions (0.53:0.47); the common disorder ratio is due to their adjacency.

Structure manipulation and figure generation were performed using Olex2.[4] Unless noted otherwise all structural diagrams containing anisotropic displacement ellipsoids are drawn at the 50 % probability level. Data collection, structure solution, and structure refinement were conducted at the X-ray Crystallographic Facility, B04 Hutchison Hall, Department of Chemistry, University of Rochester.

The crystal structure of **1** contains co-crystallized solvent molecules, namely acetone and water, that were absent from the crystal characterized in our previous report. Despite the inclusion of these solvent molecules in the structure, the geometry of the Fe_2Zn cluster obtained here is very similar to that obtained in our previous work as evidenced by the Fe-Zn representative edge distances within the triangular scaffold of cluster **1** listed in Table S2. We note that prior to using the precursor in a nanocrystal reaction, we dry the crystals at 45 ºC under vacuum. The FTIR spectrum of the dried crystalline product contains peaks associated with coordinated trifluoroacetate ligands, water and the $ZnFe_2O$ core but does not contain any peaks associated with acetone (Figure S3).

Table S1. Crystal data and structure refinement for **1.**

CCDC Deposition Number	**2172363**
Identification code	KNOKS31
Empirical formula	$C_{24}H_{32}F_{18}Fe_2O_{21}Zn$
Formula weight	1175.56
Temperature	100.00(10) K
Wavelength	1.54184 Å
Crystal system	triclinic
Space group	*P*-1
Unit cell dimensions	*a* = 10.5761(2) Å α = 92.7280(10)°
	b = 11.6114(2) Å β = 97.2040(10)°
	c= 19.8778(3) Åγ= 111.860(2)°
Volume	2235.84(7) $Å^3$
Z	2
Density (calculated)	1.746 Mg/m^3
Absorption coefficient	7.140 mm^{-1}
F(000)	1176
Crystal color, morphology	orange, needle
Crystal size	0.217 x 0.062 x 0.027 mm^3
Theta range for data collection	2.252 to 77.969°
Index ranges	$-13 \le h \le 13$, $-14 \le k \le 14$, $-25 \le l \le 23$
Reflections collected	75218
Independent reflections	9430 [*R*(int) = 0.0714]
Observed reflections	8353
Completeness to theta = 74.504°	99.7%
Absorption correction	Multi-scan
Max. and min. transmission	1.00000 and 0.41867
Refinement method	Full-matrix least-squares on F^2
Data / restraints / parameters	9430 / 282 / 753
Goodness-of-fit on F^2	1.039
Final *R* indices [*I*>2sigma(*I*)]	*R*1 = 0.0471, *wR*2 = 0.1190
R indices (all data)	*R*1 = 0.0529, *wR*2 = 0.1230
Largest diff. peak and hole	0.935 and -0.756 e.$Å^{-3}$

Table S2. Comparison of representative edge distances within the triangular scaffold of cluster **1** and a previously reported structure with the same composition but different crystallization solvent molecules.

	Identification Code	
Representative Distances	1996869[5]	2172363 (Cluster **1**)
Zn1(Fe2)-Fe1	3.3208(7)	3.3447(6)
Zn1(Fe2)-Fe3(Zn2)	3.3632(7)	3.3547(5)
Fe1-Fe3(Zn2)	3.3071(6)	3.3058(7)

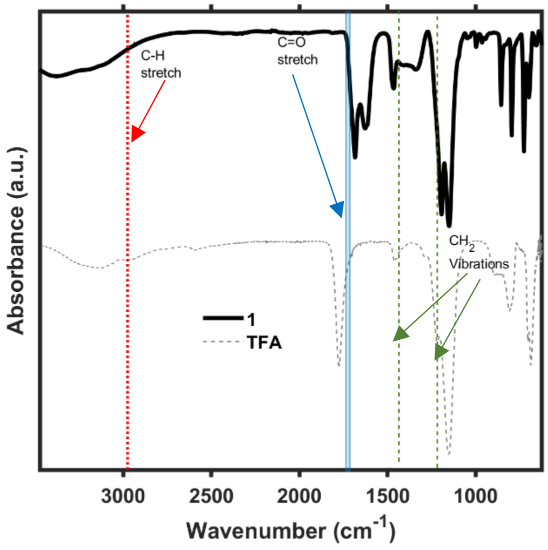

Figure S2. FTIR spectra of the Cluster 1 and free trifluoroacetic acid (TFA). The dotted lines and blue window indicate the positions of representative acetone bands that are clearly not observable in **1**.

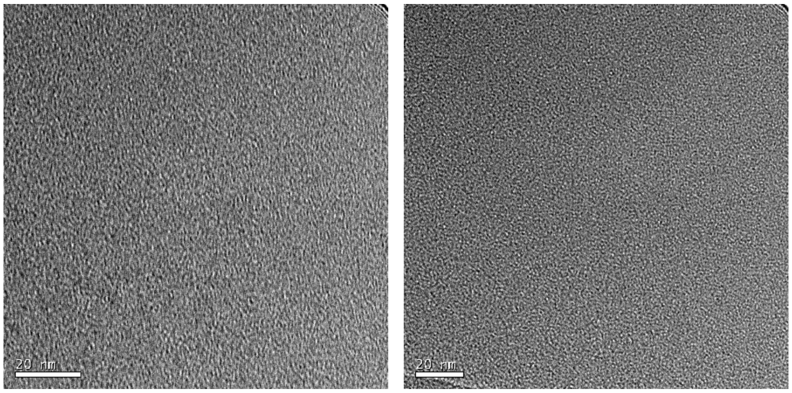

Figure S3. Representative TEM micrographs of the products obtained from hot injection (left) and heat up (right) methods carried out at a reaction temperature of 230 °C.

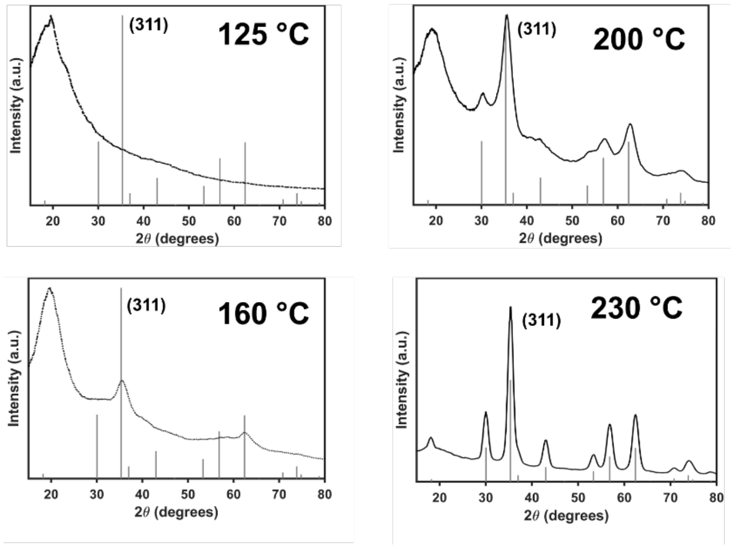

Figure S4. Powder X-ray diffraction spectra of nanoparticles obtained from solvothermal reactions of cluster precursor **1** in the presence of oleic acid, oleylamine, and benzyl ether conducted at 125, 160, 200, and 230 ºC. The crystallinity of the products improves with increasing reaction temperature, with the spinel structure fully apparent at reaction temperatures of 200 ºC and greater.

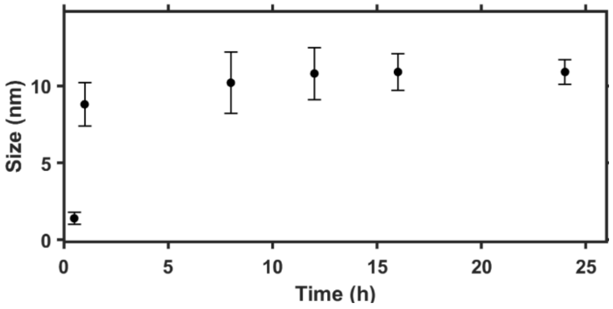

Figure S5. Plot of average diameter of $ZnFe_2O_4$ nanocrystals versus reaction time. The error bars correspond to the standard deviations of each set of at least 200 size measurements. For reaction times longer than eight hours, the average diameter remains unchanged, but the standard deviation decreases, which is indicative of size focusing.

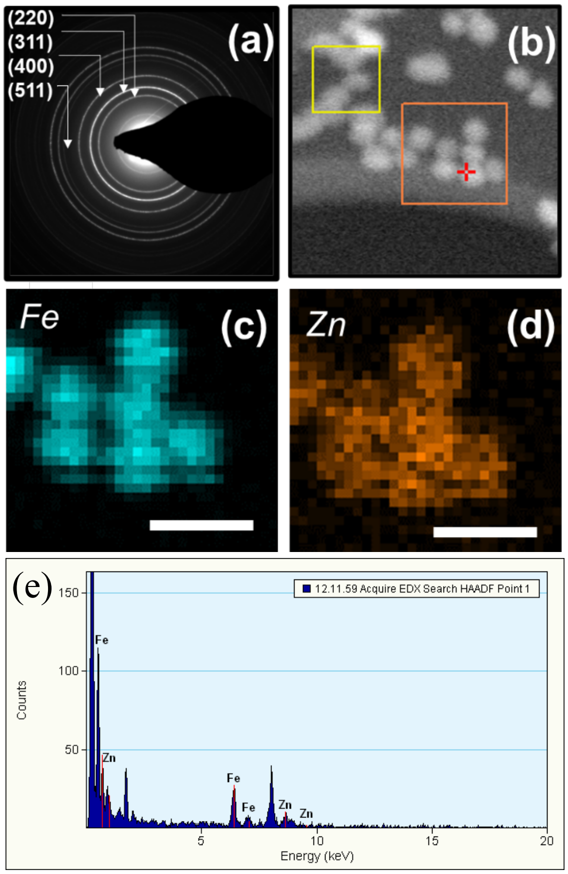

Figure S6. (**A**) Selected area electron diffraction (SAED) patterns of $ZnFe_2O_4$ nanocrystals synthesized in the presence of oleic acid, oleylamine, and benzyl ether. (**B**) STEM image of $ZnFe_2O_4$ nanoparticles from (A). (**C**) Iron Kα and (**D**) Zinc Kα elemental mapping of the particles outlined by the orange box in part A. (**E**) Integrated SEM EDX spectrum taken from the area outlined in orange in part A.

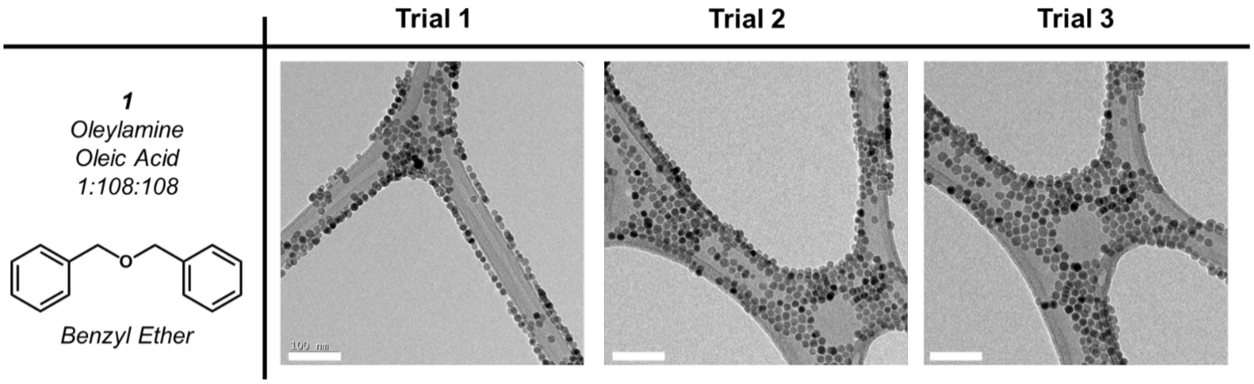

Figure S7. Representative TEM images of $ZnFe_2O_4$ nanocrystals obtained from three different reactions run under identical conditions with a **1**:OA:OAm ratio of 1:108:108 in benzyl ether at 230 ºC. The scale bars correspond to 100 nm.

Table S3. Statistical Analysis of the Diameters of $ZnFe_2O_4$ Nanocrystals Synthesized from Cluster **1** with a **1**:OA:OAm ratio of 1:108:108.[a]

Reaction Components	**Trials**			Average Diameter	Weighted STD[b]
	**1**	**2**	**3**		
**1** + OA + OAm in Benzyl Ether	10.8 ± 1	10.6 ± 1.5	11.2 ± 1.4	10.9	0.8

[a]All units in nm. [b]Calculated from equation S3.

The weighted average standard deviation was calculated from the equation S3, where σ_i and N_i correspond to the standard deviation and number of measurements, respectively, for trial *i*.

$$\langle\sigma\rangle = \frac{\sqrt{\sigma_1 {N_1}^2+\sigma_2 {N_2}^2+\sigma_3 {N_3}^2}}{N_1+N_2+N_3} \qquad \text{(S3)}$$

Table S4. Summary of reaction conditions used to vary the precursor to ligand ratio and the morphology of the resulting nanocrystals.[a]

Precursor (mmol)[b] [g]	Oleic acid (mmol)[c] [g][e]	Oleylamine (mmol)[c] [g][e]	Benzyl ether (g)[d]	Predominant Morphologies (>80%)	Diameter (nm)
0.025 [0.0231]	0.675 [0.212]	0.675 [0.185]	10.4	Spheres	6.4 ± 0.6
0.025 [0.0231]	1.35 [0.424]	1.35 [0.369]	10.4	Spheres	8.6 ± 0.4
0.025 [0.0231]	2.7 [0.848]	2.7 [0.737]	10.4	Spheres	10.9 ± 0.8
0.025 [0.0231]	5.4 [1.696]	5.4 [1.474]	10.4	Octahedrons	21.5 ± 1.8

[a.]Set focusing temperature = 230 °C, error: [b]$\pm$ 2 x10^{-4}, [c]$\pm$ 0.02, [d]$\pm$ 0.1, [e]masses account for nominal purity of oleic acid (90%) and oleylamine (98%)

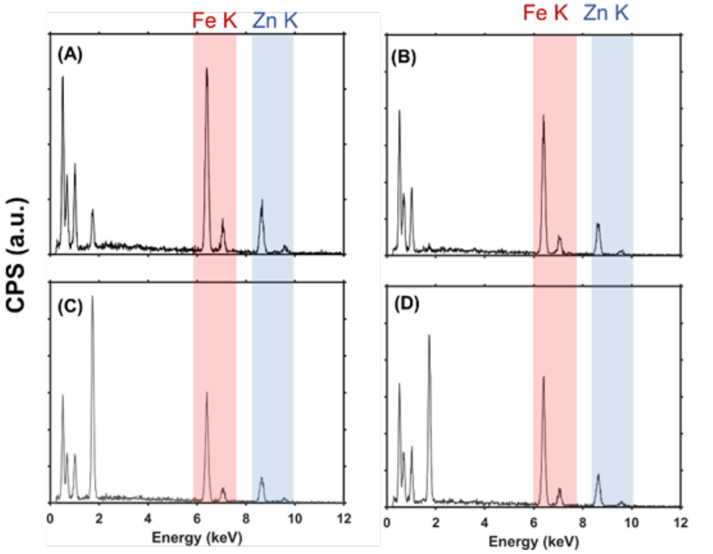

Figure S8. Energy dispersive X-ray spectra of $ZnFe_2O_4$ nanocrystals synthesized using different precursor to surfactant ligands ratios (**1**:OA:OAm). (A) 1:28:28, (B) 1:56:56, (C) 1:108:108, (D) 1:216:216.

Table S5. Atomic Percent composition of Zn and Fe in $ZnFe_2O_4$ NPs synthesized using different precursor to surfactant ligands ratios (**1**:OA:OAm) measured by EDS.

Element (keV)	1:28:28	1:56:56	1:108:108	1:216:216
Fe K (6.40)	66.71	67.36	66.72	64.07
Zn K (8.63)	33.29	32.64	33.28	35.93

Table S6. Summary of reaction conditions used to vary the ligand composition and the morphology of the resulting nanocrystals.[a]

Precursor (mmol)[b] [g]	Oleic acid (mmol)[c] [g][e]	Oleylamine (mmol)[c] [g][e]	Benzyl ether (g)[d]	Predominant Morphology (>80%)	Diameter (nm)
0.025 [0.0231]	2.7 [0.848]	2.7 [0.737]	10.43	Spheres	10.9 ± 0.8
0.025 [0.0231]	2.7 [0.848]	-	10.43	Flower-like	107 ± 10
0.025 [0.0231]	-	2.7 [0.737]	10.43	Spheres	7.8 ± 1.2
0.025 [0.0231]	-	-	10.43	Spheres	340 ± 140[f]

[a] Set focusing temperature = 230 °C, error: [b]± 2 x10^{-4}, [c]± 0.03, [d]± 0.1, [e]masses account for nominal purity of oleic acid (90%) and oleylamine (98%), [f]Size of nanocrystal aggregates

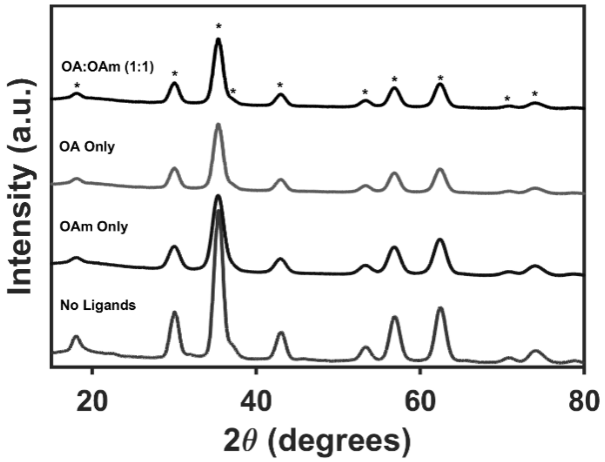

Figure S9. Powder X-ray diffraction patterns of reaction products obtained in the presence of both oleic acid and oleylamine (1:1), in the presence of oleic acid and absence of oleylamine (OA only) in the presence of oleylamine and absence of oleic acid (OAm only), in the absence of both oleic acid and oleylamine (no ligands). The * symbol indicates the position of standard reference peaks for the spinel zinc ferrite crystal phase (JCPDS Card No. 01-089-1009.)

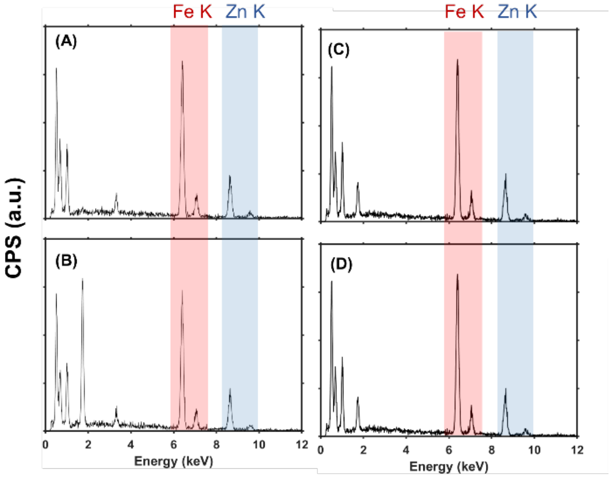

Figure S10. Energy dispersive X-ray spectra of $ZnFe_2O_4$ nanocrystals synthesized at 230 °C in benzyl ether as solvent containing different ligand mixtures. (**A**) OAm and OA in same molar ratio, (**B**) Only OA present, (**C**) Only OAm as ligand and (**D**) No ligands.

Table S7. Atomic Percent composition of Zn and Fe in $ZnFe_2O_4$ NPs Synthesized in the Presence of Different Ligand Mixtures as Measured by EDS

Element (keV)	OAm and OA in same molar ratio	Only OA present	Only OAm as ligand	No ligands
Fe K (6.40)	66.63	66.39	66.70	66.67
Zn K (8.63)	33.37	33.61	33.30	33.33

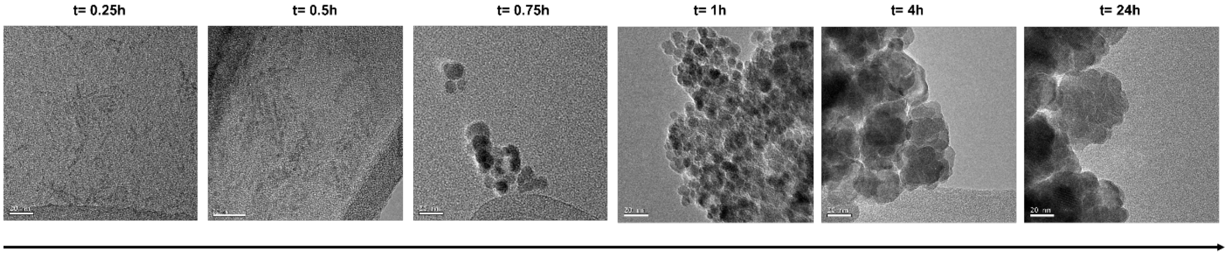

Figure S11. Representative TEM images of products obtained after various reaction times in the presence of oleic acid and absence of oleylamine. These images demonstrate that, in the absence of oleylamine, the $ZnFe_2O_4$ nanocrystals aggregate as soon as they form.

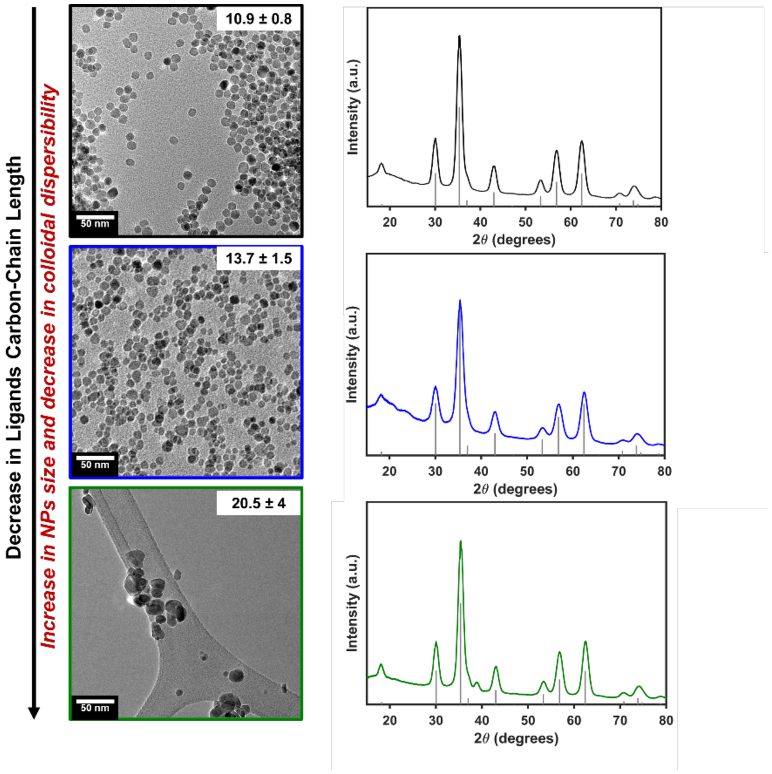

Figure S12. Representative TEM images and powder XRD spectra of $ZnFe_2O_4$ nanocrystals obtained from the reaction of 1 in the presence of carboxylic acid and amine ligands of decreasing chain lengths. (*Top*) oleic acid and oleylamine (N = 18 carbons), (*Middle*) lauric acid and lauryl amine (N = 12 carbons), and (*Bottom*) hexanoic acid and hexylamine (N = 6 carbons). The precursor to ligand ratios for all reactions were **1**:acid:amine = 1:108:108.

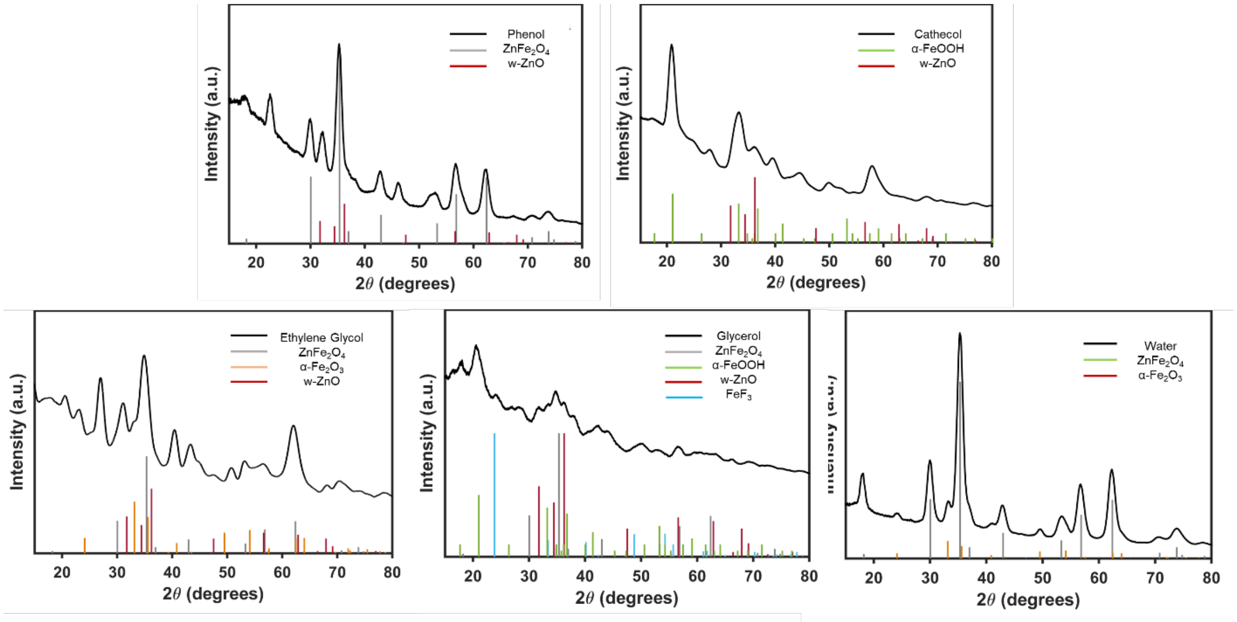

Figure S13. Powder X-ray diffraction spectra of nanocrystal samples obtained from solvothermal reactions in solvents containing hydroxyl groups. Reference peaks for the following compounds are plotted: $ZnFe_2O_4$ (JCPDS: 01-089-1009), w-ZnO (JCPDS: 00-036-1451), goethite (α-FeOOH, JCPDS: 29-0713), α-Fe_2O_3 (JCPDS: 33-0664), and FeF_3 (JCPDS: 33-0647).

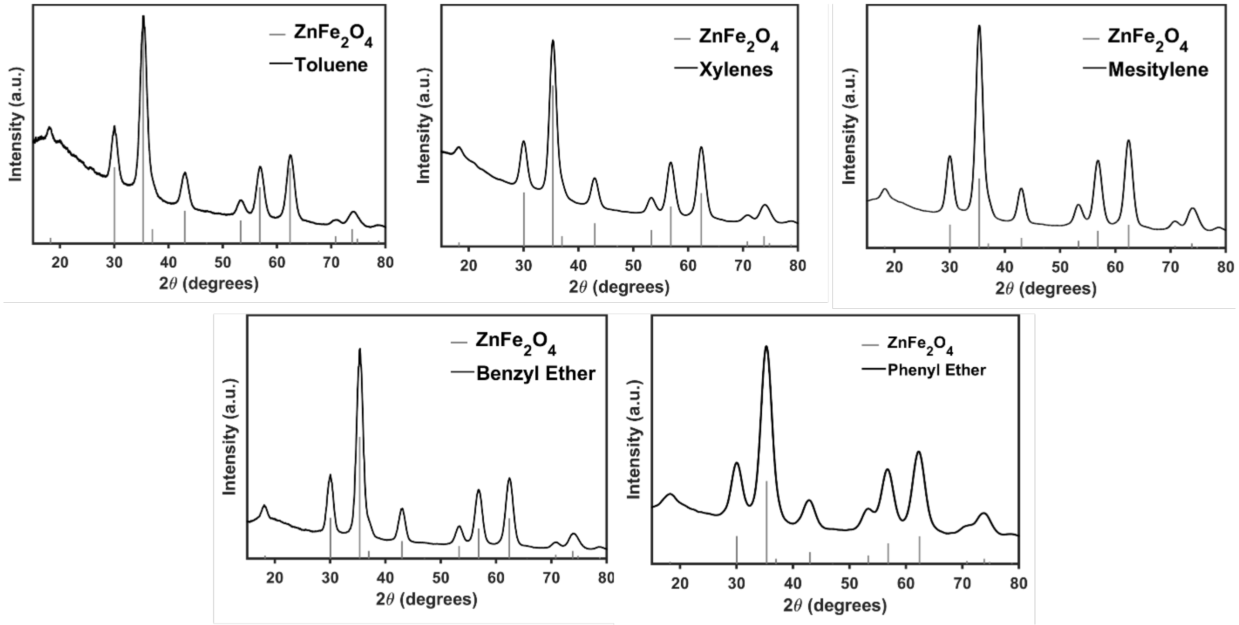

Figure S14. Powder X-ray diffraction spectra of nanocrystals obtained from solvothermal reactions in aromatic solvents that do not contain hydroxyl groups.

Table S8. Summary of reaction conditions used for various reaction solvents.

Solvent Identity	Precursor (mmol)[b] [g]	Oleic acid (mmol)[c] [g][e]	Oleylamine (mmol)[c] [g][e]	Solvent (g)[d]
Phenol	0.025 [0.0231]	2.7 [0.848]	2.7 [0.737]	10.7
Catechol	0.025 [0.0231]	2.7 [0.848]	2.7 [0.737]	13.4
Ethylene glycol	0.025 [0.0231]	2.7 [0.848]	2.7 [0.737]	11.1
Glycerol	0.025 [0.0231]	2.7 [0.848]	2.7 [0.737]	12.6
Water	0.025 [0.0231]	2.7 [0.848]	2.7 [0.737]	10.0
Toluene	0.025 [0.0231]	2.7 [0.848]	2.7 [0.737]	8.67
Xylenes	0.025 [0.0231]	2.7 [0.848]	2.7 [0.737]	8.64
Mesitylene	0.025 [0.0231]	2.7 [0.848]	2.7 [0.737]	8.63
Benzyl ether	0.025 [0.0231]	2.7 [0.848]	2.7 [0.737]	10.43
Phenyl ether	0.025 [0.0231]	2.7 [0.848]	2.7 [0.737]	10.73

[a.]Set focusing temperature = 230 °C, error: [b]± 2 x10^{-4}, [c]± 0.03, [d]± 0.1, [e]masses account for nominal purity of oleic acid (90%) and oleylamine (98%)

Lattice Parameter Calculation

The lattice parameters (a) of $ZnFe_2O_4$ nanocrystals were determined from the positions of diffraction peaks in the original powder XRD spectrum collected with the Molybdenum X-ray source, using equations S3 and S4.

$$d_{hkl} = \frac{\lambda}{2sin\theta} \quad \text{(S3)}$$

$$a = d_{hkl}\sqrt{h^2 + k^2 + l^2} \quad \text{(S4)}$$

In these equations, d_{hkl} is the d-spacing, λ is the wavelength of the X-ray source used to collect the data, θ is the Bragg angle, and *hkl* are the miller indices associated diffraction peak. We calculated values of lattice parameters from the seven most intense peaks in the XRD spectrum and report their average in Table 2 of the main test. Table S8 contains the complete set of data used in these calculations.

Table S9. Powder X-ray diffraction data used to determine the Lattice Parameter of $ZnFe_2O_4$ nanocrystals.

Phenyl Ether

λ (Å)	Miller indices			Bragg's angle		d-spacing (Å)	$\sqrt{(h^2 + k^2 + l^2)}$	Lattice constant (Å)	Average a (Å)
	h	k	l	2θ	θ				
0.71073	1	1	1	8.39	4.195	4.8579	1.732050	8.41422	8.415634
0.71073	2	2	0	13.77	6.885	2.9644	2.828427	8.38463	
0.71073	3	1	1	16.09	8.045	2.5392	3.316624	8.42161	
0.71073	4	0	0	19.42	9.71	2.1069	4	8.42789	
0.71073	4	2	2	23.87	11.935	1.7183	4.898979	8.41832	
0.71073	5	1	1	25.36	12.68	1.6189	5.196152	8.41222	
0.71073	4	4	0	27.59	13.795	1.4903	5.656854	8.43052	

Benzyl Ether

λ (Å)	Miller indices			Bragg's angle		d-spacing (Å)	$\sqrt{(h^2 + k^2 + l^2)}$	Lattice constant (Å)	Average a (Å)
	h	k	l	2θ	θ				
0.71073	1	1	1	8.41	4.205	4.846421	1.732050	8.394249	8.406556
0.71073	2	2	0	13.77	6.885	2.964415	2.828427	8.384632	
0.71073	3	1	1	16.11	8.055	2.536081	3.316624	8.411232	
0.71073	4	0	0	19.47	9.735	2.101613	4	8.406455	
0.71073	4	2	2	23.86	11.93	1.719093	4.898979	8.421803	
0.71073	5	1	1	25.37	12.685	1.618305	5.196152	8.408961	
0.71073	4	4	0	27.63	13.815	1.488204	5.656854	8.418557	

Xylenes

λ (Å)	Miller indices			Bragg's angle		d-spacing (Å)	$\sqrt{(h^2 + k^2 + l^2)}$	Lattice constant (Å)	Average a (Å)
	h	k	l	2θ	θ				
0.71073	1	1	1	8.39	4.195	4.857954	1.732050	8.414223	8.420889
0.71073	2	2	0	13.74	6.87	2.970856	2.828427	8.402851	
0.71073	3	1	1	16.07	8.035	2.542352	3.316624	8.432030	
0.71073	4	0	0	19.42	9.71	2.106972	4	8.427890	
0.71073	4	2	2	23.88	11.94	1.717674	4.898979	8.414852	
0.71073	5	1	1	25.34	12.67	1.620190	5.196152	8.418754	
0.71073	4	4	0	27.573	13.7865	1.491221	5.656854	8.435622	

Mesitylene

λ (Å)	Miller indices			Bragg's angle		d-spacing (Å)	$\sqrt{(h^2 + k^2 + l^2)}$	Lattice constant (Å)	Average a (Å)
	h	k	l	2θ	θ				
0.71073	1	1	1	8.39	4.195	4.857954	1.732051	8.414223	8.420370
0.71073	2	2	0	13.73	6.865	2.97301	2.828427	8.408942	
0.71073	3	1	1	16.08	8.04	2.540782	3.316625	8.426821	
0.71073	4	0	0	19.42	9.71	2.106973	4	8.427891	
0.71073	4	2	2	23.88	11.94	1.717675	4.898979	8.414853	
0.71073	5	1	1	25.352	12.676	1.619436	5.196152	8.414835	
0.71073	4	4	0	27.575	13.7875	1.491115	5.656854	8.435023	

Toluene

λ (Å)	Miller indices	Bragg's angle	d-spacing (Å)	$\sqrt{(h^2 + k^2 + l^2)}$	Lattice constant (Å)	Average a (Å)

λ (Å)	h	k	l	2θ	θ	d-spacing (Å)	$\sqrt{(h^2+k^2+l^2)}$	Lattice constant (Å)	Average a (Å)
0.71073	1	1	1	8.41	4.205	4.846422	1.732051	8.394249	8.409488
0.71073	2	2	0	13.73	6.865	2.97301	2.828427	8.408942	
0.71073	3	1	1	16.12	8.06	2.534519	3.316625	8.406049	
0.71073	4	0	0	19.45	9.725	2.103754	4	8.415016	
0.71073	4	2	2	23.89	11.945	1.716966	4.898979	8.411382	
0.71073	5	1	1	25.36	12.68	1.618933	5.196152	8.412223	
0.71073	4	4	0	27.63	13.815	1.488205	5.656854	8.418558	

Benzyl Ether (Only OAm)

λ (Å)	Miller indices			Bragg's angle		d-spacing (Å)	$\sqrt{(h^2+k^2+l^2)}$	Lattice constant (Å)	Average a (Å)
	h	k	l	2θ	θ				
0.71073	1	1	1	8.389	4.1945	4.858532	1.732051	8.415225	8.422845
0.71073	2	2	0	13.75	6.875	2.968706	2.828427	8.39677	
0.71073	3	1	1	16.07	8.035	2.542353	3.316625	8.432031	
0.71073	4	0	0	19.41	9.705	2.108048	4	8.432191	
0.71073	4	2	2	23.87	11.935	1.718384	4.898979	8.418327	
0.71073	5	1	1	25.3	12.65	1.62271	5.196152	8.431847	
0.71073	4	4	0	27.58	13.79	1.49085	5.656854	8.433523	

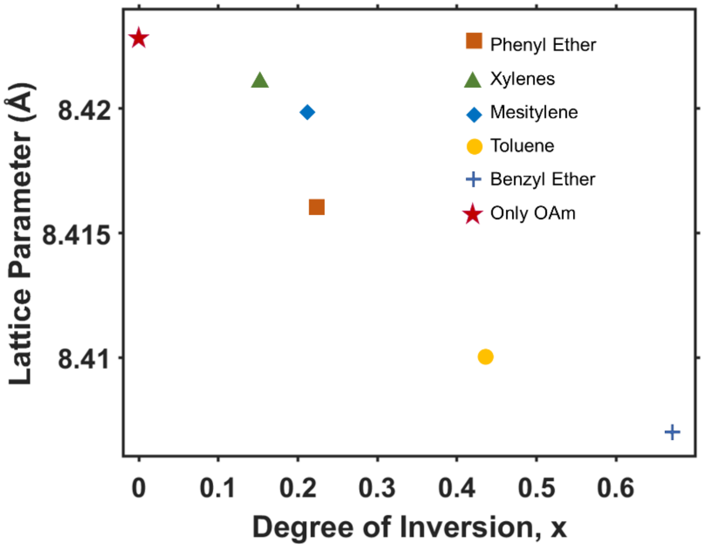

Figure S15. Plot of lattice constant determined from powder X-ray diffraction data versus the inversion parameter x for $ZnFe_2O_4$ nanocrystals synthesized in aromatic OH-free solvents.

Determination of Solubility of 1 in Various Reaction Solvents

The molar absorption coefficient of **1** was determined using four stock solutions at 25ºC diluted to absorbances between 0.1 and 1.0 in accordance with the Beer-Lambert's law. Saturated solutions of **1** were prepared by adding the solid into different solvents at 25ºC, namely benzyl ether, toluene, xylenes and mesitylene, with stirring overnight, and then allowed to settle for at least 1 h. The solutions were then filtered through glass wool to remove any undissolved material. An aliquot of each solution was diluted in its respective solvent and the UV-Vis spectra collected to determine the concentration of **1** in the saturated solution. Given miscibility issues between the solvents of interest and acetonitrile, we decided to record UV-Vis data of the resulting solutions in the solvent they were incubated for 24 hours. Therefore, it is important to note that we assumed the molar extinction coefficient of **1** @340 nm will be basically the same from solvent to solvent in order to perform the solubility analysis.

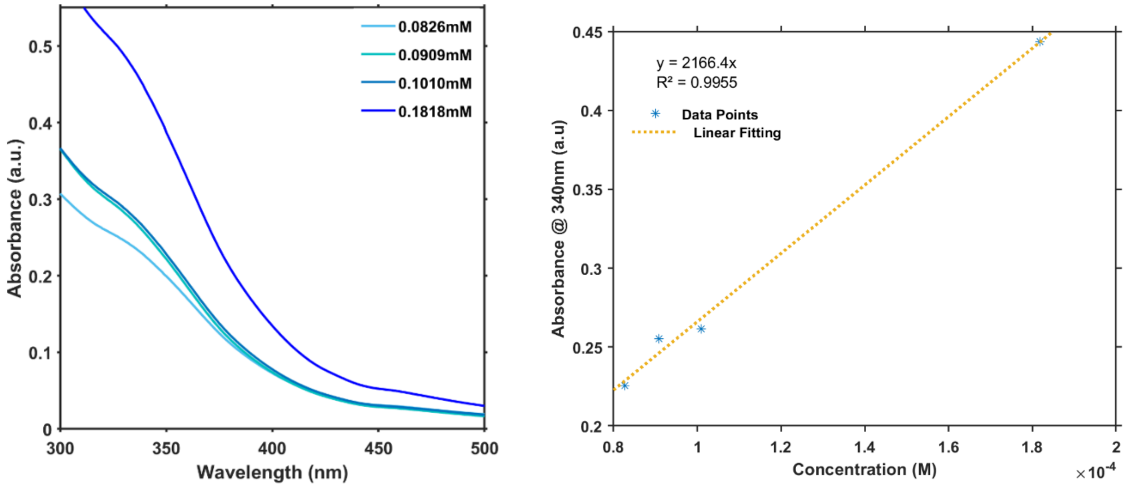

Figure S16. Absorption spectra (*left*) and associated Beer-Lambert's law plot of absorbance at 340 nm versus concentration of **1** in acetonitrile (*right*). The dotted line is a linear fit whose slope corresponds to the product of the extinction coefficient, ε, and the cell pathlength, *l*. Since *l* = 1 cm, this slope indicates that ε = 2166 $M^{-1}cm^{-1}$.

Table S10. Absorbance data used to determine the concentrations of saturated solutions of **1** in various solvents.

	Absorbance @ 340 nm	Dilute Concentration (mM)	Dilution Factor	Saturated Concentration (mM)
**1**-Benzyl Ether	0.532	0.000245	5 µL to 1000 µL	0.04911
**1**-Toluene	0.509	0.000235	10 µL to 41.0 µL	0.00097
**1**-Xylenes	0.559	-	-	0.00026
**1**-Mesitylene	0.482	0.000222	10 µL to 13.0 µL	0.00029

Table S11. Physicochemical properties of solvent media

	Cation Distribution	Density (g/mL)	Solubility (mol/L)	Dielectric constant	Enthalpy of Vaporization (kJ/mol) $\Delta_{vap}H°$*	Boiling Point
Xylenes	0.15 ± 0.5	0.864	0.00026	2.2-2.4	42	138.5
Mesitylene	0.21 ± 0.072	0.863	0.00029	2.40	47.5	164.7
Phenyl Ether	0.22 ± 0.02	1.073	0.00000	3.9	66	258
Toluene	0.4 ± 0.15	0.867	0.00097	2.38	38	110.6
Benzyl Ether	0.67 ± 0.001	1.043	0.04911	3.86	45.6	298

*NIST Chemistry WebBook

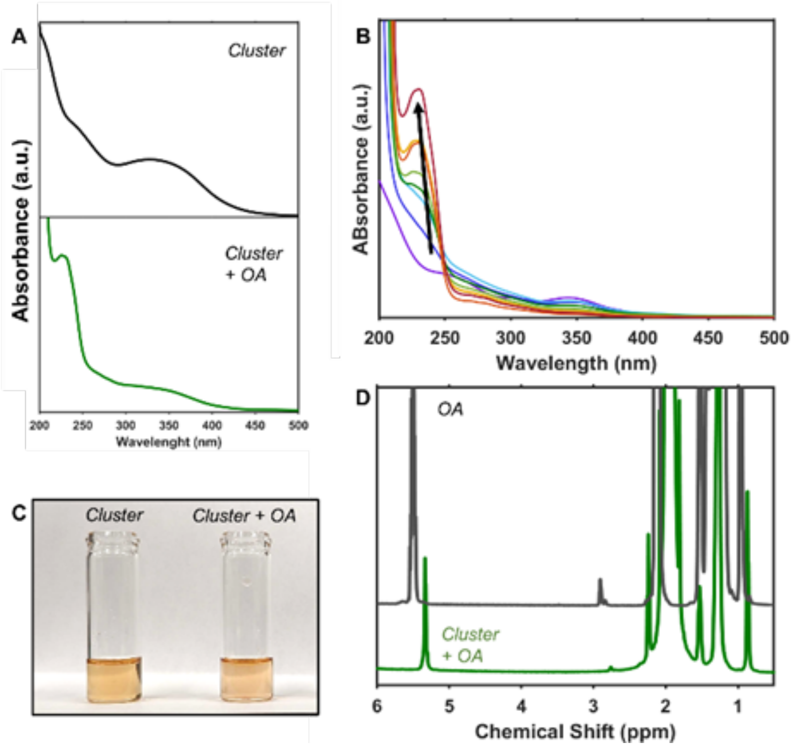

Figure S17. (**A**) Electronic absorption spectra of **1** and **1** + oleic acid in acetonitrile. (**B**) Absorption spectra of solutions of **1** and various amounts of oleic acid in acetonitrile. The initial concentration of **1** was 2.5 mM and oleic acid was added in aliquots of 0.38 mmol until a total amount of 2.7 mmol had been added. As the concentration of oleic acid increases (from purple to red), the intensity of the peak centered at λ_{max} = 240 nm increases and the intensity of the peak centered at λ_{max} = 350 nm decreases. (**C**) Photographs of the solutions characterized in (**A**). (**D**) ^{1}H-NMR spectra of solutions of OA and **1** + OA in $CDCl_3$ show a shift in the position of the peak associated with vinylic protons on the oleic acid structure (δ = 5.49 ppm for OA to δ = 5.33 ppm for **1** + OA). These shifts are indicative of ligand exchange.

Characterization of ligand exchange with cluster 1 by ^{19}F NMR

We collected ^{19}F NMR spectra to characterize the starting $ZnFe_2O$ cluster (which contains trifluoroacetate ligands), the ligand exchange reaction between the cluster and oleic acid, and the supernatant acquired after purification of the nanoparticles. These spectra are shown in Figure S18. Compared to the ^{19}F NMR spectraof free trifluoroacetic acid, which contains one narrow resonance at $\delta \sim -76$ ppm, the $ZnFe_2O$ cluster contains two broader resonances shifted to more positive chemical shifts ($\delta \sim -35$ ppm and $\delta \sim -53$ ppm). The observed broadening and shifting is consistent with bonding of the trifluoroacetate to a paramagnetic metal center (i.e., Fe^{3+}) and the observation of two peaks is consistent with the two bonding environments occupied by trifluoroacetate ligands in the cluster precursor. Four of the six trifluoroacetate ligands bridge the Zn^{2+} center and one of the Fe^{3+} centers. We assign the narrower peak at $\delta \sim -53$ ppm to these ligands. The other two trifluoroacetate ligands bridge the two Fe^{3+} centers. Bonding to two paramagnetic metal centers should increase the peak shift and peak broadening, thus we assign the broader peak at $\delta \sim -35$ ppm to these two ligands. Importantly, the spectrum of the cluster does not contain any peaks associated with free trifluoroacetic acid.

After 24 hours in the presence of oleic acid at room temperature (**1**:OA = 1:108), a narrow peak at a chemical shift similar to that of free trifluoroacetic acid appears in the spectrum alongside the peaks for the bound trifluoroacetate ligands, indicating that the addition of oleic acid displaces trifluoroacetic acid from the cluster. Addition of both oleic acid and oleylamine to the cluster (**1**:OA:OAm = 1:108:108) produces a ^{19}F NMR spectrum that contains only free trifluoroacetic acid, indicating that all of the bound trifluoroacetic acid had been displaced from the cluster. Finally, the ^{19}F NMR spectrum of the supernatant collected after purification of the nanocrystals contains a sharp doublet peak centered at a chemical shift that is slightly more negative than the free trifluoroacetic acid. We hypothesize that this feature may arise from products of the reaction of displaced trifluoroacetic acid with oleylamine (i.e. an amide) and/or with oleic acid (i.e. an acid anhydride).

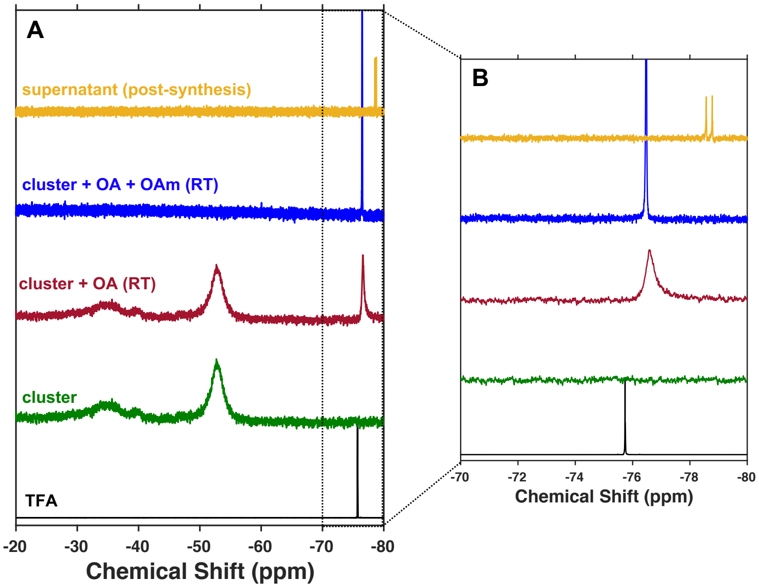

Figure S18. (**A**) ^{19}F-NMR spectra of free trifluoroacetic acid (black), the cluster precursor 1 (green), the cluster precursor after 24 hours at room temperature in the presence of oleic acid (dark red) and oleic acid and oleylamine (blue), and of the supernatant collected after purification of the nanocrystals (orange). (**B**) The same ^{19}F NMR spectra as shown in A but zoomed in to show the peaks in the region of free trifluoroacetic acid. The spectra of solutions containing cluster **1** were prepared in benzyl ether at the same concentrations used in the nanocrystal reactions. The spectrum of free trifluoroacetic acid was collected in benzyl ether.

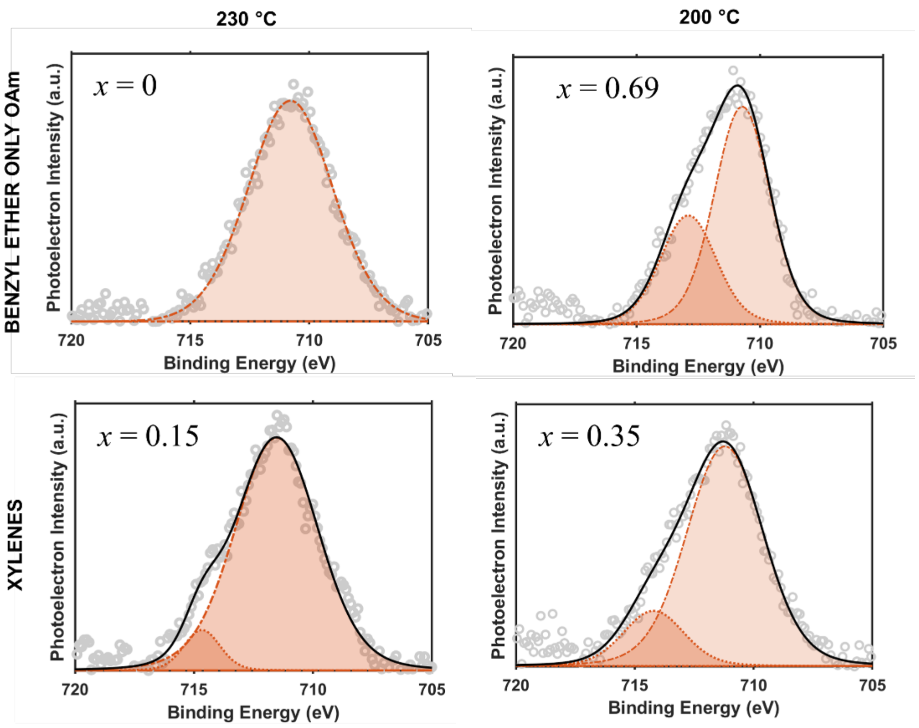

Figure S19. Plots of the Fe $2p_{3/2}$ region of the x-ray photoelectron spectra of $ZnFe_2O_4$ nanocrystals synthesized in two different reaction mixtures - in the presence of benzyl ether and oleylamine (**A, B**) or xylenes, oleic acid and oleylamine (**C, D**) and at two different reaction temperatures 230 ºC (**A, C**) and 200 ºC (**B, D**). For both reaction mixtures, decreasing the reaction temperature results in an increase in the inversion parameter, *x*.

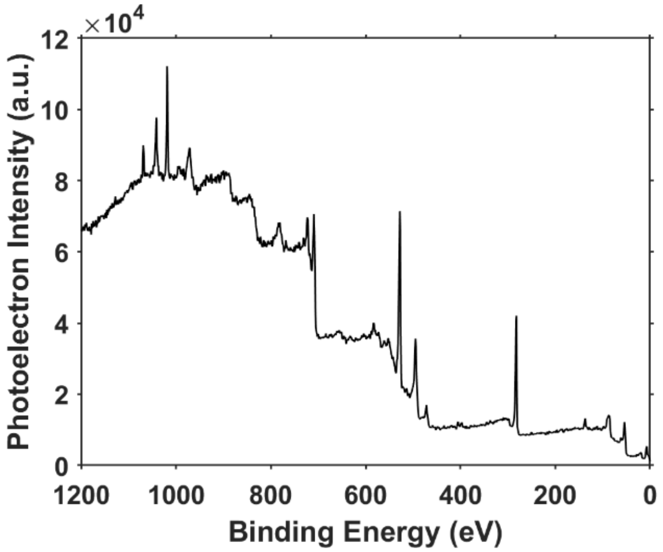

Figure S20. Representative X-ray photoelectron survey spectra of the $ZnFe_2O_4$ nanocrystals synthesized from cluster **1**. The scan indicates the presence of Zn, Fe, O, and C (from surface ligands).

Note on the impact 2+ vs 3+ metal charge has on ligand exchange/hydrolysis

There are two competing factors we consider when assessing the relative probability of hydrolysis occurring at M^{2+} versus M^{3+} sites: the ability of water to access the metal center via displacement of a carboxylate ligand (a kinetic factor) and the relative stability of the product metal hydroxide complex (a thermodynamic factor). The smaller positive charge on the M^{2+} center makes it less Lewis acidic than the M^{3+} center and results in a weaker bond to the bridging carboxylate ligands. We thus anticipate that the M^{2+} center will be more kinetically accessibly to incoming water molecules. However, we also expect the M^{2+} ion to form a weaker bond with a hydroxide ligand for similar reasons, which makes it more susceptible to the reverse reaction. Overall, we suspect that these two factors effectively cancel out, resulting in no particular preference for any metal center to be hydroxylated over any other at any given time during the reaction.

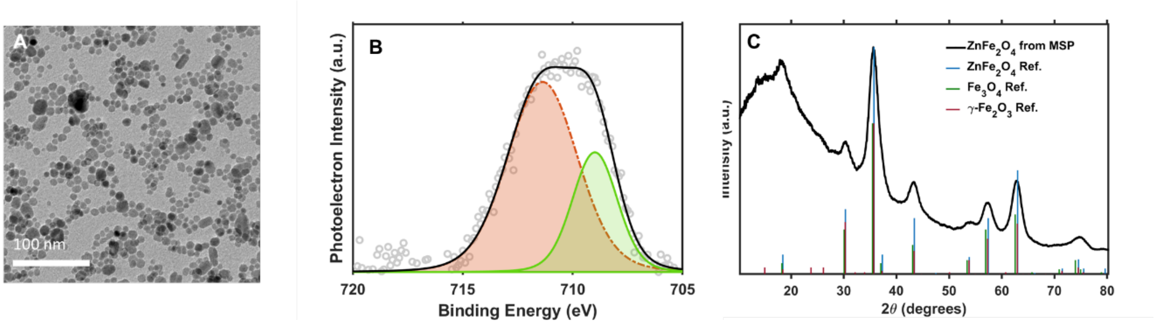

Figure S21. Characterization of the nanocrystals obtained from reaction of a 1:2 mixture of zinc(II) nitrate and iron(III) nitrate in the presence of oleic acid and oleylamine. (**A**) A representative TEM micrograph indicates the formation of a polydisperse sample of isotropic nanoparticles. (**B**) Fe $2p_{3/2}$ XPS spectra is consistent the presence of both Fe^{3+} (orange peak centered at 711.3 eV) and Fe^{2+} (green peak centered at 708.8 eV). (**C**) The powder x-ray diffraction spectrum of the resulting nanocrystals shows only peaks corresponding to spinel crystal structures. Reference spectra for $ZnFe_2O_4$ (JCPDS 01-089-1009) and Fe_3O_4 (JCPDS 19-1346) are shown to demonstrate that these structures have nearly identical powder X-ray diffraction patterns.

References

[1] *CrysAlisPro*, version 171.41.104a; Rigaku Corporation: Oxford, UK, 2021.

[2] Sheldrick, G. M. *SHELXT*, version 2018/2; *Acta. Crystallogr.* **2015**, *A71*, 3-8.

[3] Sheldrick, G. M. *SHELXL*, version 2018/3; *Acta. Crystallogr.* **2015**, *C71*, 3-8.

[4] Dolomanov, O. V.; Bourhis, L. J.; Gildea, R. J.; Howard, J. A. K.; Puschmann, H. *Olex2*, version 1.3-ac4; *J. Appl. Cryst.* **2009**, *42*, 339-341.

[5] Sanchez-Lievanos, K.S.; Tariq, M.; Brennessel, W. W; Knowles. *Dalton Trans.*, **2020**, *49*, 16348-16358.

[6] Fortune, Jodany. (2018). Re: How can we find Lattice parameter from XRD plot ?. Retrieved from: https://www.researchgate.net/post/How-can-we-find-Lattice-parameter-from-XRD plot/5bebd869979fdc494655970a/citation/download.
